# Genome-Wide Analysis of *CCT* Transcript Factors to Identify Genes Contributing to Photoperiodic Flowering in *Oryza rufipogon*

**DOI:** 10.3389/fpls.2021.736419

**Published:** 2021-11-08

**Authors:** Xin Peng, Win Tun, Shuang-feng Dai, Jia-yue Li, Qun-jie Zhang, Guo-ying Yin, Jinmi Yoon, Lae-hyeon Cho, Gynheung An, Li-zhi Gao

**Affiliations:** ^1^Institution of Genomics and Bioinformatics, South China Agricultural University, Guangzhou, China; ^2^Crop Biotech Institute, Graduate School of Biotechnology, Kyung Hee University, Yongin, South Korea; ^3^Department of Plant Bioscience, Pusan National University, Miryang, South Korea

**Keywords:** *Oryza rufipogon*, rice, *CCT* genes, genomic synteny, expression profiles, photoperiodic flowering regulation

## Abstract

Photoperiod sensitivity is a dominant determinant for the phase transition in cereal crops. *CCT* (*CONSTANS, CO-like, and TOC1*) transcription factors (TFs) are involved in many physiological functions including the regulation of the photoperiodic flowering. However, the functional roles of *CCT* TFs have not been elucidated in the wild progenitors of crops. In this study, we identified 41 *CCT* TFs, including 19 *CMF*, 17 *COL*, and five *PRR* TFs in *Oryza rufipogon*, the presumed wild ancestor of Asian cultivated rice. There are thirty-eight orthologous *CCT* genes in *Oryza sativa*, of which ten pairs of duplicated *CCT* TFs are shared with *O. rufipogon*. We investigated daily expression patterns, showing that 36 *OrCCT* genes exhibited circadian rhythmic expression. A total of thirteen *OrCCT* genes were identified as putative flowering suppressors in *O. rufipogon* based on rhythmic and developmental expression patterns and transgenic phenotypes. We propose that *OrCCT08*, *OrCCT24*, and *OrCCT26* are the strong functional alleles of rice *DTH2*, *Ghd7*, and *OsPRR37*, respectively. The SD treatment at 80 DAG stimulated flowering of the LD-grown *O. rufipogon* plants. Our results further showed that the nine *OrCCT* genes were significantly downregulated under the treatment. Our findings would provide valuable information for the construction of photoperiodic flowering regulatory network and functional characterization of the *CCT* TFs in both *O. rufipogon* and *O. sativa*.

## Introduction

*Oryza rufipogon* Griff. is widely considered as the perennial progenitor of Asian cultivated rice (*Oryza sativa* L.) and serves as promising sources of elite alleles for rice improvement (Khush, 1997; Stein et al., 2018; Zhao et al., 2018). Modern rice varieties have expanded from their primitive domesticated regions to a wide range of latitudes from 53°N to 40°S as a result of the photoperiodic diversification during rice domestication (Koo et al., 2013). In cereal crops, photoperiod sensitivity, the dominant determinant for the phase transition from vegetative growth to reproductive growth, is regulated by the interaction between endogenous circadian clocks and exogenous day lengths which varies based on the difference in geographical latitudes (Koo et al., 2013). As a result of adaptation, flowering plants have a suitable flowering time to propagate offspring by sensing the seasonal cues. When the external solar rhythm agrees with the circadian rhythm, the time signal promotes the synthesis of *CO*/*Hd1* that activates the expression of florigens which move from leaves to the shoot apical meristem (SAM) to trigger flowering (Song et al., 2015). In rice, the two flowering pathways, *OsGI*-*Hd1*-*Hd3a*/*RFT1* under short-day (SD) and *OsGI*-(*Hd1*/*Ghd7*/*DTH8*)-*Ehd1*-*H3da*/*RFT1* under long-day (LD), have been well elucidated (Hori et al., 2016; Zong et al., 2021). In addition, some flowering regulators are not involved in the two main flowering pathways, for example, *DTH2* activates flowering by directly upregulating *Hd3a* and *RFT1* (Wu et al., 2013).

*CCT* (*CONSTANS*, *CO-like*, and *TOC1*) transcription factors (TFs) that possess a conserved CCT domain are commonly present in flowering plants (Strayer et al., 2000). The *CCT* TFs can be divided into the three subfamilies depending on their domains (Li and Xu, 2017). The CCT motif (CMF) family proteins, like *Ghd7*, possess a CCT domain. The CONSTANS-like (COL) subfamily proteins, such as *CO* and *Hd1*, are characterized by one or two zinc finger B-box (BBOX) and a CCT domain. The members of pseudo-response regulator (PRR) subfamily encode a response-regulator (REC) domain at the N-terminus and the CCT domain at the C-terminus (Cockram et al., 2012). *CCT* genes regulate photoperiodic flowering, circadian rhythms, vernalization as well as defense against abiotic stresses (Zhang J. et al., 2015; Omolade et al., 2016; Li and Xu, 2017; Liu et al., 2020). It was reported that eighteen rice *OsCCT* genes are involved in flowering regulation (Zhang et al., 2020). *Hd1*, *Ghd2, Ghd7, OsCCT1, OsCOL4, OsCOL10*, and *DTH7* inhibited the expression of *Ehd1* under LD. Under SD, the expression of *Ehd1* is suppressed by *OsCO3*, *OsCOL4*, and *OsCOL10*, while *Hd1* and *DTH2* induce *Ehd1* (Li and Xu, 2017). In addition, *OsCCT3*, *OsCCT22*, *OsCCT38*, and *OsCCT41* were found as flowering regulators (Zhang et al., 2020).

As the wild progenitor of Asian cultivated rice, *O. rufipogon* has attracted great attention to investigating population genetics, adaptation, speciation, and gene flow (Morishima et al., 1961; Gao and Hong, 2000; Gao et al., 2001; Gao, 2002, 2004; Zheng and Ge, 2010; Huang et al., 2012; Stein et al., 2018; Li et al., 2020a; Xie et al., 2020; Xu et al., 2020). Our previous investigation suggested that natural populations of *O. rufipogon* exhibited clinal variation in flowering time from north to south within its range in China (Gao et al., 2000). The *CCT* TFs have been identified and functionally elucidated in several crop species, such as rice (Zhang et al., 2017), maize (Huang et al., 2018), wheat (Yan et al., 2004), barley (Turner et al., 2005), and *Medicago truncatula* (Ma et al., 2020). However, functional roles of *CCT* TFs have not been elucidated in their wild progenitors, such as *O. rufipogon* in this study. It is widely recognized that *O. rufipogon* has very strong photoperiod sensitivity for flowering, which inhibits flowering under LD and induces flowering only under SD (Zong et al., 2021). But its response to photoperiod remains to be investigated in *O. rufipogon*.

In this study, we performed a genome-wide identification of the *OrCCT* TFs in *O. rufipogon*. Our results showed that, under LD, most *OrCCT* genes displayed rhythmic expression and regulated flowering time as suppressors. We also found that, compared with *O. sativa*, *O. rufipogon* plants took nearly double time for vegetative growth to reach the point when the plants can respond to the SD-induction to induce flowering. Our findings presented here would provide valuable information for the construction of photoperiod response, flowering regulatory network, and functional characterization of the *CCT* gene family in both *O. rufipogon* and *O. sativa*.

## Materials and Methods

### Materials and Growth Conditions

*Oryza sativa* ssp. *japonica* cv. *Nipponbare* and *O. rufipogon* (named CWR1) which were collected from Yuanjiang County, Yunnan Province, China, were studied in this study (Li et al., 2020a). They both display photoperiod sensitivity, in which flowering is delayed under LD conditions and induced under SD conditions. Seeds were germinated on ½ Murashige and Skoog medium for 10 days. Seedlings were transplanted to plastic pots and grown in the controlled growth room under either LD (14/10 light/dark cycle, 28/22°C) or SD (10/14 light/dark cycle, 28/22°C) conditions. Light intensity was approximately 1,000 μmol m^–2^ s^–1^ with humidity of approximately 50%.

### Identification of *CCT* Transcription Factors

The two genome assemblies of *O. rufipogon* (Li et al., 2020b) and *Nipponbare* (Ouyang et al., 2007) were retrieved to identify *CCT* TFs. The *Nipponbare* reference genome (RGAP_7) was downloaded from RGAP database^1^. The longest isoforms were extracted using the Fast Get Representative program of TBtools^2^. Unless otherwise stated, the longest isoform was used throughout the study. HMMER 3.0 was employed to screen the protein sets with the Hidden Markov Model (HMM)^3^ file of CCT (PF06203), BBOX (PF00643), and REC (PF00072) as queries (cutoff = 0.01, other parameters of default). The putative CCT proteins in which the length of the aligned domain is smaller than 50% of what HMM file annotated were filtered out. The redundant sequences were discarded after BLASTP searches (*E*-value < 10^––10^). Proteins containing CCT domain and lacking BBOX and REC domains were classified as *CMF* genes. Proteins with CCT domain and additional BBOX or REC domain toward their amino-terminus were defined as *COL* or *PRR* genes, respectively. The deduced *CCT* TFs were further checked for the existence of the corresponding domain by using the Conserved Domain Database^4^. We named the *CCT* TFs with initials of genus and species and numerical symbols based on their chromosomal locations.

The molecular weight (D) and isoelectric point (Pi) of OrCCT TFs were calculated by ExPASy^5^. The web-server BUSCA was used to predict the subcellular localization of OrCCT proteins^6^. The information of position on chromosomes, exons, introns, and UTR regions of *OrCCT* genes was extracted from the gene finding format (GFF3) file. MEME software^7^ was used to identify the conserved motifs with the width of each motif = 10–100 amino acid residues, maximum number of motifs = 10, and other parameters of default values (Bailey et al., 2009). The visualization of gene structure and conserved domain (including classification) were conducted using the Gene Structure View tool of TBtools (Chen et al., 2020).

### Identification of Orthologous *CCT* Genes Between *O. rufipogon* and *O. sativa*

Multiple Collinearity Scan toolkit (MCScanX) is often used to scan multiple genomes to detect putative homologous chromosomal regions using genes as anchors (Wang et al., 2012). To identify the putative orthologous *CCT* genes between *O. rufipogon* and *O. sativa*, the inter-species collinear relationship was identified using MCScanX with the parameters recommended by MCScanX’s manual (Wang et al., 2012). The collinear and syntenic gene pairs of *CCT* genes were extracted from the MCScanX output files. In this step, the data sets include both paralogs and orthologs. To remove the possible paralogs, the genes that showed the same order on chromosomes were selected as orthologous *CCT* genes between *O. rufipogon* and *O. sativa*.

Gene duplication events within *CCT* TFs were detected by MCScanX (Wang et al., 2012), and then visualized by Advanced Circos software (see text footnote 2). Non-synonymous (*ka*) and synonymous (*ks*) substitution of the paired *CCT* genes were calculated using KaKs_Calculator 2.0 (Wang et al., 2010). Gene duplication events were approximately dated according to the eq. *T* = *Ks*/2λ (λ = 6.5 × 10^–9^) (Yu et al., 2005). The comparative synteny relationships of *CCT* TFs between *O. rufipogon* and *O. sativa* were constructed by Multiple Synteny Plotter software (see text footnote 2).

### Phylogenetic Analysis

All identified *CCT* TFs were divided into the three subfamilies according to their domains. The sequence of *CCT* TFs from *Brachypodium distachyon* and *O. sativa* ssp. *indica* was downloaded from the Phytozome database v13^8^. The sequence of *OnCCT* TFs was downloaded from the Gramene database^9^. Multiple sequence alignment of CCT full proteins from the four species was performed by using MAFFT 7.243 with E-INS-i algorithm (Katoh and Standley, 2013). The Neighbor-Joining (NJ) phylogenetic tree was inferred by MEGA6 (Kumar et al., 1994) with bootstraps = 1,000.

### RNA-Sequencing and Data Analyses

Total RNA was extracted from the leaves of 90-day-old plants using the QIAGEN plant RNA kit (Hilden, Germany). The concentration and quality of RNA were evaluated using NanoDrop 2000 UV-VIS spectrophotometer (NanoDrop Technologies, Wilmington, DE, United States). Paired-end reads were generated on a HiSeq 2000 platform following the manufacturer’s instructions (Illumina, United States). RNA-sequencing (RNA-seq) data were mapped on the reference genome with HISAT2 2.1.0 (Kim et al., 2019). FeatureCounts 1.6.2 was used to count the number of reads mapped on exons (Liao et al., 2014). Differentially expressed genes (DEGs) were evaluated by edgeR 3.32.0 (Robinson et al., 2010). Genes with *p* < 0.05 and log2 fold-changes >1 were considered as DEGs. Further screening among the initial DEGs was performed based on fragments per kilo-base per million fragments mapped (FPKM) values.

### RNA Isolation and Quantitative Real-Time PCR

Total RNAs were extracted from the leaves using RNAiso Plus (TaKaRa, Shiga, Japan). The first cDNA strand was synthesized with 2 μg total RNA, using Moloney murine leukemia virus reverse transcriptase (Promega, Madison, WI, United States) with 10 ng of the oligo(dT) 18 primer and 2.5 mM deoxyribonucleotide triphosphate. Synthesized cDNAs were used as templates for quantitative real-time PCR (qRT-PCR) with SYBR Premix Ex Taq II (TaKaRa) and the Rotor-Gene 6000 instrument system (Corbett Research, Sydney, NSW, Australia). The primers used for qRT-PCR were designed according to *O. rufipogon* reference sequences. The specificity of primers in both *O. rufipogon* and *Nipponbare* was checked by melting curve. The relative expression levels were calculated with rice *Ubi1* as an internal control. Each dataset was collected from five independent biological repeats. The primers used are listed in Supplementary Table 1.

### Vector Construction and Transformation

The 2,427-bp full-length genome DNA sequence of *OrCCT24* was amplified from CWR1 using PCR with specific primers (CATAAGCTTTATCCGTTCATGTCGATGGGA and CC GGTACCCTATCTGAACCATTGTCCAAGC, where underlined sequences indicate *Hin*dIII and *Kpn*I enzyme sites, respectively). The PCR fragments were cloned into the pGEM-T Easy vector for blue-white screening. After checking the insert by DNA sequencing, the cloned fragment from the positive clone was moved into the overexpression binary vector pGA3426 under the control of the maize *ubiquitin 1* promoter (Kim et al., 2009). After checking its quality by DNA-sequencing, the recombinant vector was transformed into *Nipponbare* via *Agrobacterium*-mediated co-cultivation (An et al., 1989). Transgenic rice plants were generated through the stable transformation method as previously reported (An et al., 1989). The putative positive calli were transferred to shoot induction medium that contains 40 mg L^–1^ hygromycin.

## Results

### Identification, Classification, and Structure of *CCT* Transcription Factors

**FIGURE 1:**
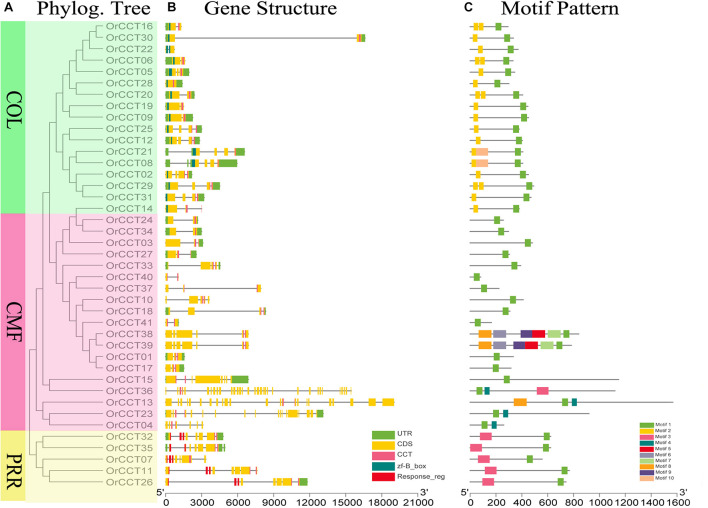
Phylogenetic relationship, gene structure, and conserved motifs of *OrCCT* TFs in *O. rufipogon*. **(A)** Phylograms of *OrCCT* TFs were constructed based on the full-length protein sequences. Different subfamilies are highlighted with different colors. *PRR* in yellow, *COL* in green, and *CMF* in red. **(B)** Exon-intron structure and conserved domains of OrCCT TFs. **(C)** The motif patterns of OrCCT proteins. The sequence information for each motif is given in Supplementary Table 3.

We identified 41 candidate *OrCCT* TFs in *O. rufipogon* (PRJCA002637)^10^. The proteins were named as OrCCT01 to OrCCT41 according to their chromosomal locations (Figure 1 and Supplementary Table 2). In addition, 41 *OsCCT* TFs were identified in the *Nipponbare* reference genome, as previously reported (Zhang et al., 2017). The molecular weight of OrCCT proteins ranged from 9,689.86 D (OrCCT40) to 171,328.16 D (OrCCT13). Their isoelectric points varied from 4.09 (OrCCT20) to 11.44 (OrCCT40) (Supplementary Table 2). Our results suggest that OrCCT proteins varied greatly among molecular features. Our prediction using BUSCA (Savojardo et al., 2018) suggested that 32 OrCCTs were located in the nucleus, while others were in chloroplast (5), extracellular space (3), and mitochondrion (1) (Supplementary Table 2).

The phylogram of *CCT* genes in *O. rufipogon* showed that *OrCCT* TFs were grouped into the three clusters based on their conserved domains (Figure 1A). The first cluster was the *CMF* subfamily with 19 members, the second was the *COL* subfamily with 17 members, and the third was the *PRR* subfamily with five members. The number of the possessed exons ranged from 1 (*OrCCT36*) to 33 (*OrCCT28*) (Figure 1B). The motif number of *OrCCT* genes alternated from 1 to 6. All *CCT* members possessed motif 1. The *CMF* members, *OrCCT38* and *OrCCT39*, had the most motif, which possessed additional motif 5, 6, 7, 8, and 9. *COL* members had additional motif 2, while motif 10 was specifically presented in *OrCCT08* and *OrCCT21*. *PRR* members possessed motif 1 and motif 3 (Figure 1C). The results suggest that the classification of *OrCCT* genes is coincident with their conserved motifs. The sequence information for each motif was present in Supplementary Table 3.

### Chromosomal Distribution, Synteny, and Evolutionary Analysis of *CCT* Genes in *O. rufipogon* and *O. sativa*

**FIGURE 2:**
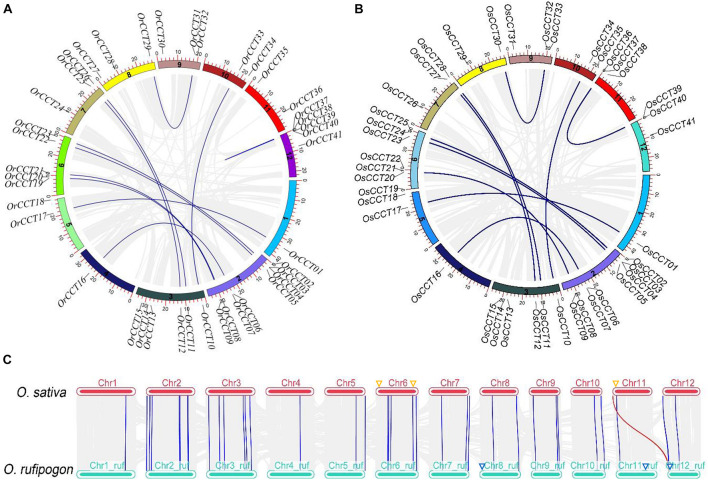
The inter-chromosomal relationship among *CCT* genes. **(A,B)** The chromosome distribution and gene duplication events in *O. rufipogon* **(A)** and *O. sativa* **(B)**. The approximate location of each *CCT* gene is marked on corresponding chromosomes. The blue lines indicate the duplicated *OrCCT* genes, and gray lines in the background represent all duplication blocks within genomes. **(C)** The collinear relationship of *CCT* genes between *O. rufipogon* and *O. sativa.* The blue lines indicate orthologous gene pairs, while the red line shows that the *CCT* gene was likely generated after the domestication of *O. sativa*. The specific *CCT* genes of *O. sativa* and *O. rufipogon* are marked with orange and blue triangles on the corresponding positions of chromosomes, respectively.

Our results showed that the *OrCCT* genes were unevenly distributed on the 12 chromosomes of *O. rufipogon*. Chromosome 1 contained the largest number of *OrCCT* TFs (8), and chromosomes 1 and 4 had only one *OrCCT* TF (Figure 2A). The distribution of *OsCCT* genes on chromosomes is similar to that in *O. rufipogon* (Figure 2B). Our results showed that there were 11 duplicated *OrCCT* gene pairs in *O. rufipogon* (Figure 2A). *OrCCT37*, *OrCCT38*, *OrCCT39*, and *OrCCT40* were present as tandem duplicated genes on Chromosome 12 (Figure 2A). Thirty-eight *OrCCT* genes had the orthologous genes in *O. sativa* (Figure 2 and Supplementary Table 4). However, the orthologs of *OrCCT27*, *OrCCT36*, and *OrCCT40* were absent in *O. sativa* (Figure 2C). In addition, we failed to identify orthologs of *OsCCT19*, *OsCCT25*, and *OsCCT37* in *O. rufipogon*, indicating that they are likely *O. sativa*-specific (Figure 2 and Supplementary Table 4).

Ten duplicated gene pairs were present in both *O. rufipogon* and *O. sativa* (Figure 2 and Supplementary Table 5). We used the formula *T* = *Ks*/2λ to evaluate approximate dates of duplicated genes (DEs). The dates of shared DEs of *CCT* genes varied from 23.05 to 89.31 million years ago (Mya) in *O. rufipogon* and *O. sativa* (Supplementary Table 5). The DE *OsCCT37*-*OsCCT40*, which was estimated to generate about 0.7 Mya, was absent in *O. rufipogon*, indicating that it probably occurred after the domestication of *O. sativa*. ω (*dN*/*dS*) is a good indicator of selective pressure at both nucleotide and protein levels. It is often expected that ω > 1, ω = 1, and ω < 1 imply positive selection, neutral selection, and purifying selection, respectively (Zhang et al., 2014). Our results suggest that nearly all duplicated *CCT* gene pairs underwent negative selection in both *O. rufipogon* and *O. sativa* (Supplementary Table 5).

**FIGURE 3:**
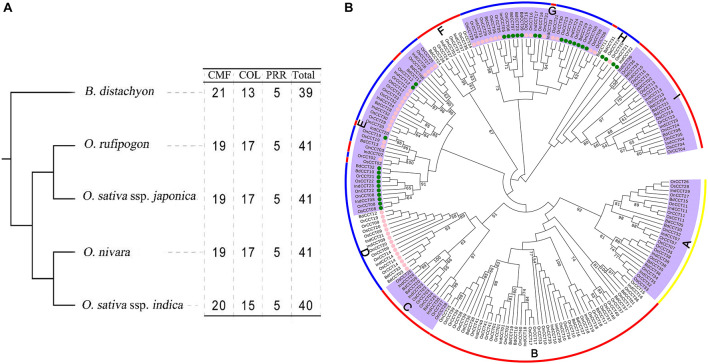
Phylogenetic relationships of *CCT* TFs among *B. distachyon*, *O. sativa* ssp. *japonica*, *O. sativa* ssp. *indica*, *O. nivara*, and *O. rufipogon*. **(A)** The species tree following the number of *CCT* TFs among the investigated species; **(B)** Phylogenetic tree representing relationships among *CCT* TFs from the four plant species. The pink and green circles of the terminal node indicate *COL* gene with 1 and 2 BBOX, respectively. The prefixes of tree labels are Bd, *B. distachyon*; Os, *O. sativa* ssp. *japonica*; Ind, *O. sativa* ssp. *indica*; On, *O. nivara*, and Or, *O. rufipogon*. The subfamilies are marked with red line: *CMF*; blue: *COL*; yellow: *PRR*. The locus of CCT TFs presenting here is listed in Supplementary Table 6.

We further investigated the diversification of CCT TFs in *Brachypodium distachyon*, *Oryza nivara*, and *O. sativa* ssp. *indica* (Supplementary Table 6). All investigated species possessed five *PRR* genes. Our results showed that *O. rufipogon*, *O. nivara*, and *O. sativa* ssp. *japonica* possessed the same composition of *CCT* subfamilies (19 *CMF*, 17 *COL*, and five *PRR*) (Figure 3A and Supplementary Table 6). A phylogenetic tree of *CCT* TFs was constructed using the complete protein sequences from the four species, including *B. distachyon*, *O. rufipogon*, *O. nivara*, *O. sativa* ssp. *japonica*, and *O. sativa* ssp. *indica*. As shown in Figure 3B, the CCT proteins could be divided into three clusters with nine clades (A to I). Clade A contained all PRR proteins. Clade B, C, F, and I consisted of CMF sub-family proteins. Clade D possessed COL proteins. The clade E, G, and H were composed of COL and few CMF proteins. Interestingly, the COL proteins were closely related to CMF proteins, suggesting that the COL proteins might originate from CMF proteins by gaining the BBOX domain. Alternatively, the CMF proteins were derived from COL proteins due to the loss of the BBOX domain.

### Daily Expression Profiling of *OrCCTs* in Long-Day and Short-Day Conditions

Rice senses the day length by endogenous genetic factors to onset reproductive growth (Cho et al., 2017). Previous results suggested that 18 *OsCCT* genes are flowering regulators in *O. sativa* (Zhang et al., 2020). To investigate whether *OrCCT* can respond to photoperiod, the *O. rufipogon* plants were grown under LD and SD, and the second leaves from the top of main stems were collected at Zeitgeber time (ZT)-2 h, 8 h, and 15 h at 90 days after germination (DAG), respectively. RNA-seq experiments generated temporal expression profiles of all 41 *OrCCT* genes with three independent replicates.

**FIGURE 4:**
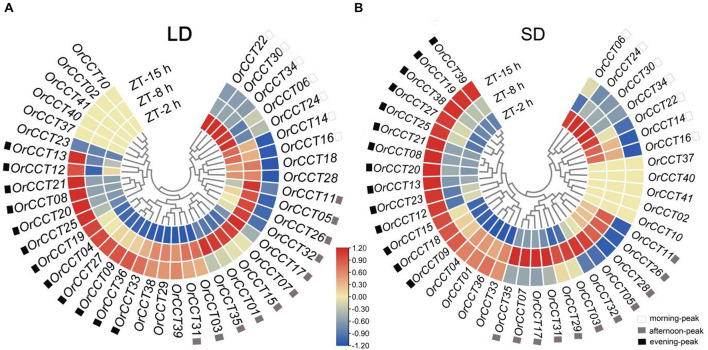
The daily expression profiles of *OrCCT* genes under LD **(A)** and SD conditions **(B)** at 90 DAG in *O. rufipogon* (CWR1). The heatmaps were drawn by FPKM values with row scale normalization (*n* = 3). The prefix ZT-2 h, ZT-8 h, and ZT-15 h indicate 2 h, 8 h, 15 h ZT, respectively.

Our results showed that thirty *OrCCT* genes showed significantly different expression levels among ZT-2 h, ZT-8 h, and ZT-15 h under LD (Figure 4A). Seven genes (*OrCCT06*, *OrCCT14*, *OrCCT16*, *OrCCT22*, *OrCCT24*, *OrCCT30*, and *OrCCT34*) were highly expressed at ZT-2 h and weakly expressed at ZT-15 h, suggesting that they are morning-peak genes. *Ghd7*, the rice ortholog of *OrCCT24*, was highly expressed in the morning (Xue et al., 2008). Twelve genes (*OrCCT04*, *OrCCT08*, *OrCCT09*, *OrCCT12*, *OrCCT13*, *OrCCT19*, *OrCCT20*, *OrCCT21*, *OrCCT025*, *OrCCT027*, *OrCCT33*, and *OrCCT36*) were highly expressed at ZT-15 h and weakly at ZT-2 h and ZT-8 h, suggesting that they are evening peak genes (Figure 4A). *OrCCT20* is the ortholog of rice *Hd1* that is highly expressed in the evening (Cho et al., 2018). Eleven genes (*OrCCT01*, *OrCCT03*, *OrCCT05*, *OrCCT07*, *OrCCT11*, *OrCCT15*, *OrCCT17*, *OrCCT26*, *OrCCT31, OrCCT32*, and *OrCCT35*) exhibited a high expression level at ZT-8 h compared to ZT-2 h and ZT-15 h (Figure 4A).

Under SD, daily expression patterns of *OrCCT* genes were similar to those observed from LD (Figure 4B). All seven genes that were expressed most highly at ZT-2 h also showed a similar morning-peak expression under SD. Among 12 evening-peak genes, nine exhibited similar daily expression patterns between LD and SD. However, three genes (*OrCCT04, OrCCT33*, and *OrCCT36*) were similarly expressed at ZT-8 h and ZT-15 h under SD conditions. Instead, five genes (*OrCCT15, OrCCT18, OrCCT23*, *OrCCT38*, and *OrCCT39*) that failed to show evening-peak under LD displayed a high expression at ZT-15 h under SD. It is well known that ZT-15 h is at the beginning of the dark period under LD whereas the time is at near midnight under SD. Thus, the difference in some *CCT* genes might be due to the day-length.

To validate the veracity of our RNA-seq results, we tested twenty *OrCCT* TFs that showed rhythmic expression by using qRT-PCR experiments. The relative expression patterns of the selected genes were almost consistent with those of RNA-seq analysis (Supplementary Figure 1).

### Developmental Expression Profiling of *OrCCT* Genes Under Long-Day Condition

**FIGURE 5:**
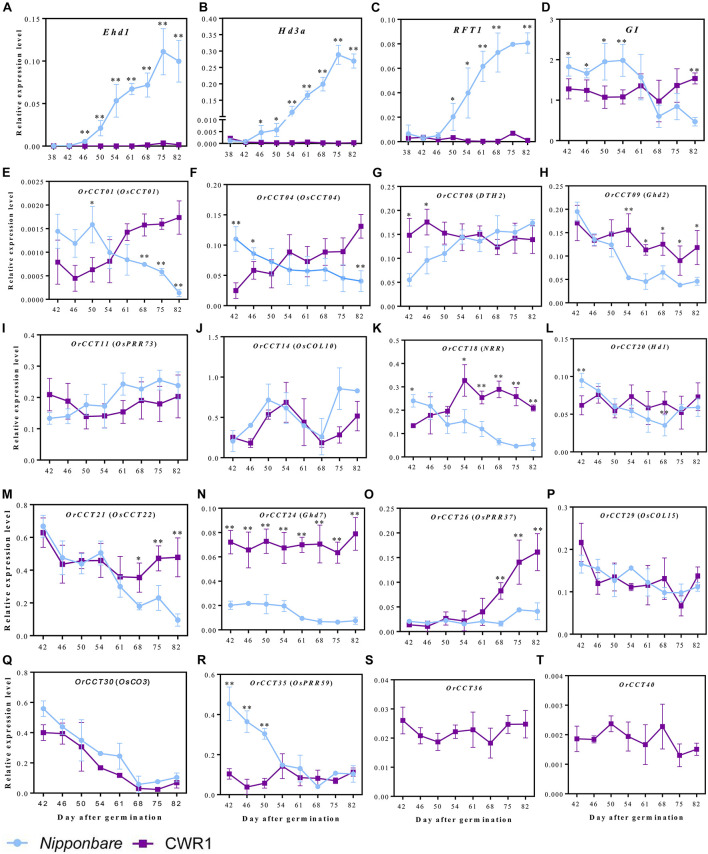
The transcript levels of *Ehd1* **(A)**, *Hd3a* **(B)**, *RFT1* **(C)**, *OsGI* **(D)**, and 16 *CCT* genes **(E–T)** in leaf blades of *Nipponbare* and *O. rufipogon* (CWR1) at different developmental stages. Leaf blade samples were isolated at ZT-2 h, ZT-8 h, and ZT-15 h at 4 days intervals starting from 38 DAG. Transcript levels are relative to *OsUbi1*. Error bars indicate standard deviation for five biological replicates. ^∗^, ^∗∗^ significant differences by Student’s *t*-test at *P* ≤ 0.05 and *P* ≤ 0.01, respectively.

The expression patterns of 16 *OrCCT* and 14 *OsCCT* genes were measured by qRT-PCR at different developmental stages under LD. Four flowering regulators, *Ehd1*, *Hd3a*, *RFT1*, and *OsGI*, were included to monitor the developmental stages of plants. The penultimate leaves of the main stems were sampled from *O. rufipogon* (CWR1) and *Nipponbare* plants at ZT-2 h, ZT-8 h, and ZT-15 h at 4 days intervals. The time points for qRT-PCR corresponded to the expression peak as shown in Figure 4. In *Nipponbare*, the transcript level of *Ehd1* rapidly started to increase at 46 DAG, peaking at 75 DAG (Figure 5A). *Hd3a* and *RFT1* also exhibited similar expression patterns with *Ehd1* in *Nipponbare* plants (Figures 5B,C). However, all three genes did not express at a detectable level during the experimental period in CWR1 (Figures 5A–C). *OsGI* kept a high expression level until 54 DAG and then rapidly declined in *Nipponbare*, while it remained at a high level in CWR1 (Figure 5D). The phenotypic observation showed that *Nipponbare* flowered at 86–90 DAG, while CWR1 showed a non-flowering phenotype when grown for >213 DAG. Our results indicate that *Nipponbare* can complete the floral transition with the promotion of *Ehd1*, *Hd3a*, and *RFT1* under LD. However, *O. rufipogon* remained at the vegetative growth phase during the investigated period.

The transcript level of *Hd1* that is a major photoperiod-sensitive floral regulator stayed at a relatively constant level in *Nipponbare* (Figure 5L). A similar expression pattern was observed for *OrCCT20* that is an ortholog of *Hd1* in *O. rufipogon*. The expression level of *Ghd7* decreased to a low level at 61 DAG after floral transition in *Nipponbare*, but the transcript amount of *OrCCT24* remained at a much high level and did not decline during the experimental period in CWR1 (Figure 5N). Similarly, the transcript levels of *Ghd2* and *OsCCT22* decreased after floral transition in *Nipponbare*, while their orthologs in *O. rufipogon*, *OrCCT09* and *OrCCT21*, respectively, remained at relatively high levels during the investigated stages (Figures 5H,M). The content of *OsPRR59* was high before the floral transition and the level declined after floral transition in *Nipponbare*, but its ortholog, *OrCCT35*, was lowly expressed at all stages in CWR1 (Figure 5R).

Three *CCT* genes interestingly exhibited opposite expression patterns between CWR1 and *Nipponbare* plants. The transcript levels of *OsCCT01*, *OsCCT04*, and *NRR* decreased as the *Nipponbare* plants grew up, whereas gene expression levels of their orthologs, *OrCCT01*, *OrCCT04*, and *OrCCT18*, increased during the experimental period in CWR1 (Figures 5E,F,K). The expression level of *OsPRR37* was relatively low and slightly increased after 75 DAG in *Nipponbare*, but the level of its ortholog *OrCCT26* increased rapidly after 61 DAG in *O. rufipogon* (Figure 5O), suggesting that *OrCCT26* is a strongly functional allele of *OsPRR37*. Transcript level of *DTH2* that is a rice flowering activator gradually increased after floral transition in *Nipponbare*, while *OrCCT08* remained at a relatively high level during the experiment in CWR1 (Figure 5G).

Several genes showed similar expression patterns between *Nipponbare* and *O. rufipogon*. The transcript levels of both *OsCO3* and *OrCCT30* were high at 42 DAG and declined to low levels at 68 DAG in *Nipponbare* and *O. rufipogon* (Figure 5Q). Expression levels of *OsPRR73* (*OrCCT11*) and *OsCOL15* (*OrCCT29*) did not vary significantly during the experimental period in both *Nipponbare* and CWR1 (Figures 5I,P). The developmental expression pattern of *OsCOL10*, a floral repressor downstream of *Ghd7* (Tan et al., 2016), was similar to its ortholog *OrCCT14* (Figure 5J), indicating that they may function similarly.

The transcript levels of *O. rufipogon*-specific *CCT* genes, *OrCCT36* and *OrCCT40*, were at a relatively low level and did not change significantly during the experimental period, indicating that they may not involve in controlling flowering (Figures 5S,T). Sequence similarity and developmental expression patterns suggest that *OrCCT08*, *OrCCT24*, and *OrCCT26* are the functional alleles of *DTH2*, *Ghd7*, and *OsPRR37*, respectively. Expression levels of *OrCCT01*, *OrCCT04*, *OrCCT09*, *OrCCT18*, *OrCCT21, OrCCT24*, and *OrCCT26* were high during the vegetative phase, suggesting that they may function as the flowering suppressor in *O. rufipogon* under LD.

### Effects of Short-Day Treatment on Flowering Time in Rice and Its Wild Progenitor

**FIGURE 6:**
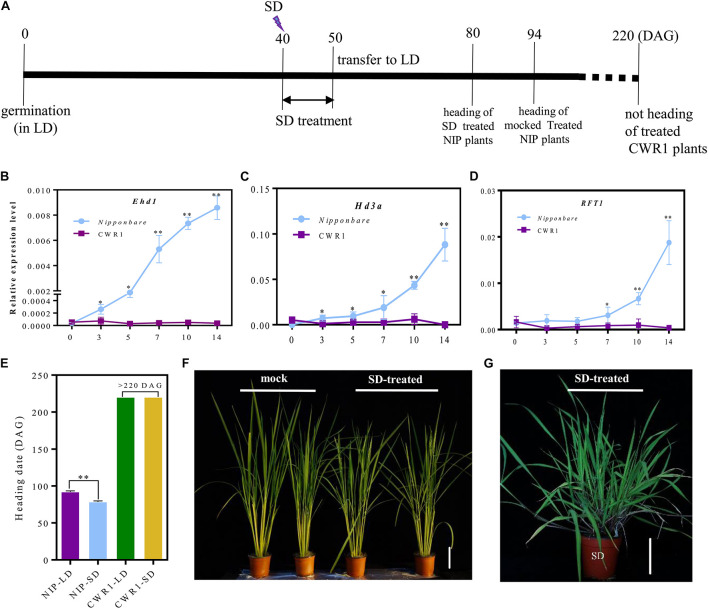
The photoperiod response to 10 days SD-treatment from 40 DAG to 50 DAG in *Nipponbare* and *O. rufipogon* (CWR1) plants. **(A)** Scheme for SD treatment. **(B–D)** The expression pattern of *Ehd1*, *Hd3a*, and *RFT1* in the plants of *Nipponbare* and *O. rufipogon* (CWR1) with 10 days SD-treatment. The *y*-axis shows the relative expression levels of genes with rice Os*Ubi1* as an internal control; the *x*-axis presents the day of SD treatment. Values are means ± SD (*n* = 5). *, ** significant differences by student’s *t*-test at *P* ≤ 0.05 and *P* ≤ 0.01, respectively. **(E)** The heading date for LD-grown plants and SD-treated plants. **(F)** Phenotypes of mock (left) and SD-treated *Nipponbare* plants (right). **(G)** The phenotype of CWR1 with 10 days SD-treatment at 80 DAG. Bar = 10 cm in **(F,G)**.

Flowering is induced by 1 week SD treatment in *O. sativa* (Doi et al., 2004). To examine whether SD treatment induces flowering in *O. rufipogon*, we applied SD treatment to LD-grown CWR1 with *Nipponbare* as a control. At 40 DAG, rice plants were transferred to the SD growth room. After 10 days treatment, these plants were transplanted back to the LD growth room until flowering (Figure 6A). All SD-treated *Nipponbare* plants flowered evenly 13.5 days earlier than the mock-control (continuously grown under LD) plants, suggesting that, as expected, 10 days SD treatment induced flowering in *Nipponbare* (Figures 6A,E,F). However, *O. rufipogon* plants treated in the same way did not induce flowering even at 180 days after the treatment (Figures 6A,E,G). For the *Nipponbare* plants, *Ehd1* and *Hd3a* were induced after 3 days of SD treatment, and the transcript level of *RFT1* increased after 7 days of treatment (Figures 6B–D). However, these three genes were expressed at low levels in the SD-treated *O. rufipogon* plants (Figures 6B–D).

**FIGURE 7:**
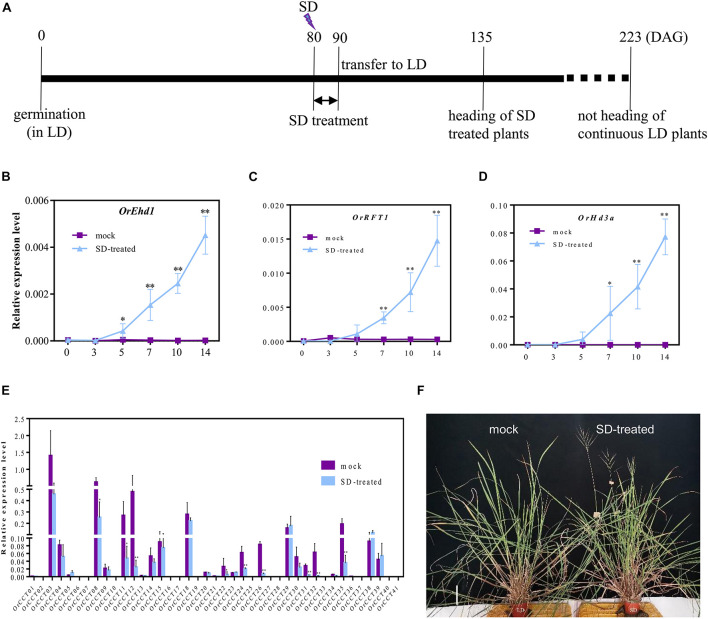
The response to SD-induction starting from 80 DAG in *O. rufipogon* (CWR1). **(A)** Scheme of SD treatment; **(B–D)** The expression level of *OrEhd1* **(B)**, *OrRFT1* **(C)**, and *OrHd3a* **(D)** in the leaf blades of CWR1 plants with mock and SD-treatment. The *x*-axis presents the days of SD treatment. **(E)** The transcript level of 41 *OrCCT* genes in the leaf blades of *O. rufipogon* (CWR1) after 10 days of SD-treatment. The transcript levels were relative to *OsUbi1*. Values are means ± SD (*n* = 5). *, ** significant differences by Student’s *t*-test at *P* ≤ 0.05 and *P* ≤ 0.01, respectively. **(F)** The phenotypes of *O. rufipogon* (CWR1) plants with mock and SD-treatment (right) at 140 DAG. Bar = 10 cm in **(F)**.

Because SD treatment at 40 DAG did not induce the expression of flowering regulatory genes, we assumed that *O. rufipogon* requires a longer vegetative growth period than *Nipponbare* before the onset of phase transition. Therefore, SD treatment was imposed on the 80-DAG CWR1 plants that were first grown under LD (Figure 7A). The SD-treated *O. rufipogon* plants flowered at 132–140 DAG, while mock plants did not flower even after growing for >223 DAG (Figures 7A,F). Compared with the mock plants, the expression of *OrEhd1* was induced after 5 days of SD treatment (Figure 7B). Similarly, transcript levels of *OrRFT1* and *OrHd3a* were increased with the treatment (Figures 7C,D).

The expression levels of 41 *OrCCT* genes further showed that, compared with mock plant, the expression of *OrCCT08*, *OrCCT11*, *OrCCT12*, *OrCCT22*, *OrCCT24*, *OrCCT26*, *OrCCT31*, *OrCCT32*, and *OrCCT35* were significantly downregulated in the SD-treated plants, indicating that these *CCT* genes function as flowering suppressors in *O. rufipogon* (Figure 7E). It was reported that *DTH2*, the orthologous gene of *OrCCT08*, may induce flowering under LD. The expression level of *DTH2* peaked at the beginning of the dark period and gradually reduced after that time (Wu et al., 2013). ZT-15 h is at the beginning of dark in LD, whereas the time is 5 h after dark in SD treatment. Therefore, the significantly decreased expression of *OrCCT08* in SD-treated plants might be due to the change of day-length.

### Effect of Overexpressed *OrCCT24* on Flowering Time Under Long-Day Condition

**FIGURE 8:**
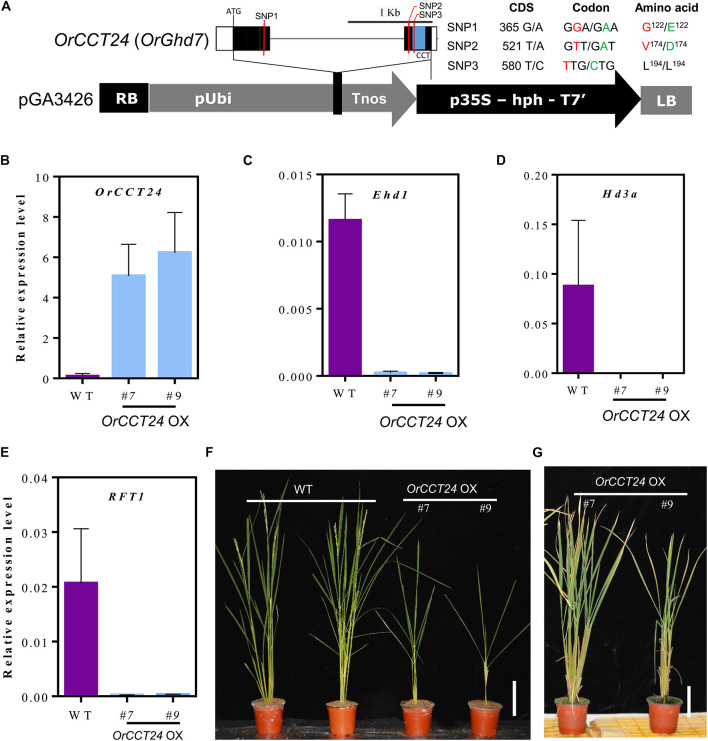
Expression and phenotype analyses of *OrCCT24*-overexpression plants under LD condition. **(A)** Scheme of *OrCCT24* overexpressed vector. **(B–E)** The expression level of *OrCCT24* **(B)**, *Ehd1* **(C)**, *Hd3a* **(D)**, and *RFT1* **(E)** in WT and *OrCCT24*-overexpressed plants. The transcript levels were relative to *OsUbi1*. Error bars indicate standard deviations (*n* = 5). Leaf blades were harvested at ZT-2 h at 60 DAG. **(F)** Phenotypes of *OrCCT24*-overexpressed plants (T0 generation) compared with WT at 90 DAG. **(G)** Phenotypes of *OrCCT24* overexpressed plants at 220 DAG. Scale bar = 10 cm.

*OrCCT24*, the ortholog of rice *Ghd7*, was highly expressed in *O. rufipogon* compared to *Ghd7* in *Nipponbare* (Figure 5N). Sequence analysis showed that three single nucleotide polymorphisms (SNPs) were found in the coding region of *OrCCT24* in CWR1 compared with *Nipponbare*. Among them, two SNPs caused amino acid substitutions, and one SNP was synonymous mutation (Figure 8A). To examine whether the high expression of *OrCCT24* caused late flowering in *O. rufipogon*, we constructed the overexpressed *OrCCT24* vector, and then transformed it into *Nipponbare* (Figure 8A). From 15 independently transformed plants, two lines with high levels of expression of *OrCCT24* were selected (Figure 8B). The developmental expression patterns showed that the expression levels of *Ehd1*, *Hd3a*, and *RFT1* rapidly increased from 50 DAG to 75 DAG in *Nipponbare*. Hence, the transcript levels of the three genes were measured at ZT-2 h from the transgenic plants at 60 DAG under LD. qRT-PCR experiments showed that the expression of *Ehd1*, *Hd3a*, and *RFT1* was induced in the wild type (WT), whereas their expression was strongly suppressed in the overexpressed *OrCCT24* plants (Figures 8C–E). The transgenic plants did not flower up to 220 DAG, while their WT controls flowered at 85–90 DAG (Figures 8F,G). Our results indicated that *OrCCT24* is a strong inhibitor of flowering by suppressing the expression of *Ehd1*, *Hd3a*, and *RFT1* in *O. rufipogon*.

## Discussion

### Flowering Regulation of *OrCCT* Genes in *O. rufipogon*

Crop wild relatives play an extremely important role in crops’ adaptation to farming practices, market demands, and climatic conditions (Dempewolf et al., 2017). Over the past decade, the reference genomes of approximately 15 *Oryza* species have been deciphered, which have greatly facilitated comprehensive allele mining in these *Oryza* species (Zhang et al., 2014; Stein et al., 2018; Li et al., 2020b; Shi et al., 2020). There have been great successes in introducing desired traits from wild rice into cultivated rice, such as cytoplasmic male sterile source (Lin and Yuan, 1980). In this study, we obtained *OrCCT* genes with the strategy of the reference genome-based gene family identification, which is time-efficient compared with traditional methods of genetic mapping (Peng et al., 2019).

In rice and *Arabidopsis*, the *PRR* subfamily is a crucial component of feedback loops of the core oscillator for the circadian clock (Mizuno and Nakamichi, 2005). In the present study, the expression levels of *PRR* TFs (*OrCCT07*, *OrCCT11*, *OrCCT26*, *OrCCT32*, and *OrCCT35*) was significantly divergent among ZT-2 h, 8 h, and 15 h both under LD and SD in *O. rufipogon* plants, suggesting that *OrPRR* genes are relevant to the circadian clock. In rice, two florigens, *Hd3a* and *RFT1*, activate floral transition by inducing the expression of *MADS14 and MADS15* (Shrestha et al., 2014). The *OsGI*-*Ghd7*-*Ehd1*-*RFT1*/*Hd3a* pathway regulates rice flowering under LD. In this pathway, *Ghd7* represses the cereal-specific flowering inducer gene *Ehd1*, thereby delays flowering by decreasing expression of *Hd3a* and *RFT1* (Doi et al., 2004; Xue et al., 2008; Cho et al., 2016). *OsPRR37*, *OsCCT01*, *Ghd2* negatively regulate flowering by downregulating *Ehd1* (Koo et al., 2013; Zhang L. et al., 2015; Liu et al., 2016). *DTH2* activates flowering by directly upregulating *Hd3a* and *RFT1* (Wu et al., 2013). Overexpressed *NRR* decreases the expression of *Hd3a* and *RFT1*, which consequently delays flowering (Zhang et al., 2013). In addition, *Hd1* suppresses flowering under LD when functional *Ghd7* is present (Fujino et al., 2019; Zhang et al., 2019).

**FIGURE 9:**
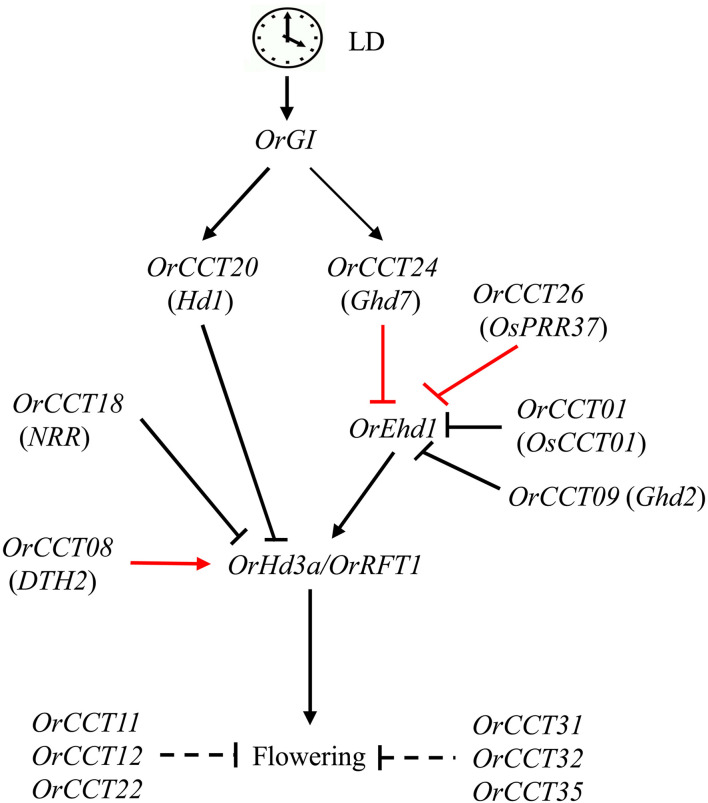
*CCT* TFs involved regulatory network for flowering time of *O. rufipogon* under LD. The clock at the top designates the circadian clock. Black arrows represent induction, and black bars indicate suppression. Red arrows show strong induction, and red bars denote strong suppression. The virtual line shows indirect effect.

In the present study, a typical *O. rufipogon* accession (CWR1) did not flower up to 213 DAG under LD condition. The developmental expression profiles revealed that the orthologs of *Ehd1* and florigens were not expressed in CWR1 under LD condition. Several *CCT* genes, including *OrCCT01*, *OrCCT04*, *OrCCT09*, *OrCCT18*, *OrCCT21*, *OrCCT24*, and *OrCCT26*, were highly expressed in CWR1 compared to *Nipponbare*, suggesting that they are repressors of flowering in *O. rufipogon*. Among these genes, orthologs of six *OrCCTs* except for *OrCCT04* are flowering suppressors in rice (Xue et al., 2008; Koo et al., 2013; Zhang et al., 2013; Zhang L. et al., 2015; Zhang et al., 2020; Liu et al., 2016). In addition, the expression of *OrCCT08*, *OrCCT11*, *OrCCT12*, *OrCCT22*, *OrCCT24*, *OrCCT26*, *OrCCT31*, *OrCCT32*, and *OrCCT35* were significantly downregulated in the SD-treated plants. With the combination of previous findings and our obtained results in this study, we propose a model for flowering regulation of *OrCCT* TFs in *O. rufipogon* under LD (Figure 9). In the model, the florigen genes *OrHd3a* and *OrRFT1* are induced by *OrEhd1* that are repressed by *OrCCT01*, *OrCCT09*, *OrCCT24*, and *OrCCT26*. Among the repressors, *OrCCT24* and *OrCCT26* are the strongest suppressors. *OrGI* positively controls *OrCCT20* and *OrCCT24* expression. In addition, *OrCCT11*, *OrCCT12*, *OrCCT22*, *OrCCT31*, *OrCCT32*, and *OrCCT35* may negatively regulate flowering (Figure 9). However, their up- and down-stream genes are still unknown. Further efforts are thus needed to elucidate the roles of *OrCCT* TFs under SD.

*OrCCT24*, the ortholog of *Ghd7*, was highly expressed during all examined developmental stages in CWR1 under LD compared to *Nipponbare*, suggesting that *OrCCT24* is a strong repressor of flowering in *O. rufipogon*. We observed that *OrCCT24* was highly expressed in the *OrCCT24* over-expressed plants compared with WT. However, the transgenic plants did not flower up to 220 DAG. *RFT1* is a major florigen that functions to induce reproductive development in the SAM. It is documented that overexpression of *RFT1* resulted in the direct formation of spikelets from most of the transgenic calli (Pasriga et al., 2019). In all, we confirm that 13 *OrCCT* TFs have played important roles in controlling flowering time, but functional roles of other *OrCCT* genes remain largely unknown.

### Photoperiod Sensitivity of *O. rufipogon*

Crops are distinguished from their wild progenitors by some typical alterations, such as the loss of seed dormancy and shattering mechanisms, reduced branching, increased fruit or seed size, and changes in photoperiod sensitivity (Olsen and Wendel, 2013). The growth of *O. rufipogon* is limited to tropical regions (Gao, 2004; Zhao et al., 2013). In this study, *O. rufipogon* plants did not flower under LD conditions, which is indicative of its high photoperiod sensitivity. In most well-known examples, the members of *CCT* TFs are involved in the adaptation for photoperiod and flowering, including *Tof11* and *Tof12* in soybean (Lu et al., 2020), as well as *Hd1*, *Ghd7*, and *OsPRR37* in rice (Koo et al., 2013; Zong et al., 2021). Our findings suggest that the daily expression patterns of 36 *OrCCT* genes (of a total of 41 members) changed with the circadian rhythm, indicating that they can respond to the light signal. We also found that 13 *OrCCT* genes are likely the flowering suppressors based on their expression patterns, and *OrCCT08*, *OrCCT24*, and *OrCCT26* serve as the strong functional alleles of rice *DTH2*, *Ghd7*, and *OsPRR37*, respectively. *Ghd7* and *OsPRR37* are the pivotal determinants for strong photoperiod sensitivity in rice (Koo et al., 2013; Zhang J. et al., 2015; Zong et al., 2021). As discussed above, we conclude that the 13 *OrCCT* TFs have likely contributed to the strong photoperiod sensitivity in *O. rufipogon*, resulting in extremely delayed flowering under LD.

Rice cultivation has been expanded from its primitive domesticated regions to wide regions due to the long-term natural and artificial selection during rice domestication and subsequent modern improvement (Fujino et al., 2013). In this study, 10 days SD-treatment at the early developmental stage (40 DAG) did not promote flowering in *O. rufipogon*. Such a result indicates that the wild rice plants may require a long vegetative growth stage before responding to SD induction. When 80 days LD-grown plants were treated with 10 days of SD-induction, the treated *O. rufipogon* plants flowered at 52–60 days after the treatment while untreated control plants did not flower. During the SD treatment, nine *OrCCT* genes were significantly downregulated, indicating that they can respond to SD to regulate flowering in *O. rufipogon*. The photoperiod-responding *OrCCT* genes may be applied to breeding new rice varieties that possess higher biomass and increased grain yields.

## Data Availability Statement

The datasets presented in this study can be found in online repositories. The names of the repository/repositories and accession number(s) can be found below: National Genomics Data Center, accession no: PRJCA006767.

## Author Contributions

L-ZG and GA conceived and designed the study. XP, WT, S-FD, J-YL, Q-JZ, G-YY, JY, and L-HC conducted the experiments. XP and WT analyzed the data. XP wrote the draft of the manuscript. L-ZG, XP, and GA revised the manuscript. All authors read and approved the manuscript.

## Conflict of Interest

The authors declare that the research was conducted in the absence of any commercial or financial relationships that could be construed as a potential conflict of interest.

## Publisher’s Note

All claims expressed in this article are solely those of the authors and do not necessarily represent those of their affiliated organizations, or those of the publisher, the editors and the reviewers. Any product that may be evaluated in this article, or claim that may be made by its manufacturer, is not guaranteed or endorsed by the publisher.

## Footnotes

1
http://rice.uga.edu/

2
https://github.com/CJ-Chen/TBtools

3
http://pfam.xfam.org/

4
https://www.ncbi.nlm.nih.gov/cdd/

5
https://web.expasy.org/protparam/

6
http://busca.biocomp.unibo.it/

7
http://meme-suite.org/tools/meme

8
https://phytozome.jgi.doe.gov/

9
http://archive.gramene.org/

10
https://bigd.big.ac.cn/gsa/
