# Supplementary material for: Genome-Wide Analysis of CCT Transcript Factors to Identify Genes Contributing to Photoperiodic Flowering in Oryza rufipogon
Source: Front Plant Sci. 2021 Nov 8;12:736419. doi: 10.3389/fpls.2021.736419 (PMC8606741; doi:10.3389/fpls.2021.736419)
Supplement: Supplementary file 1 [file Data_Sheet_1.docx]

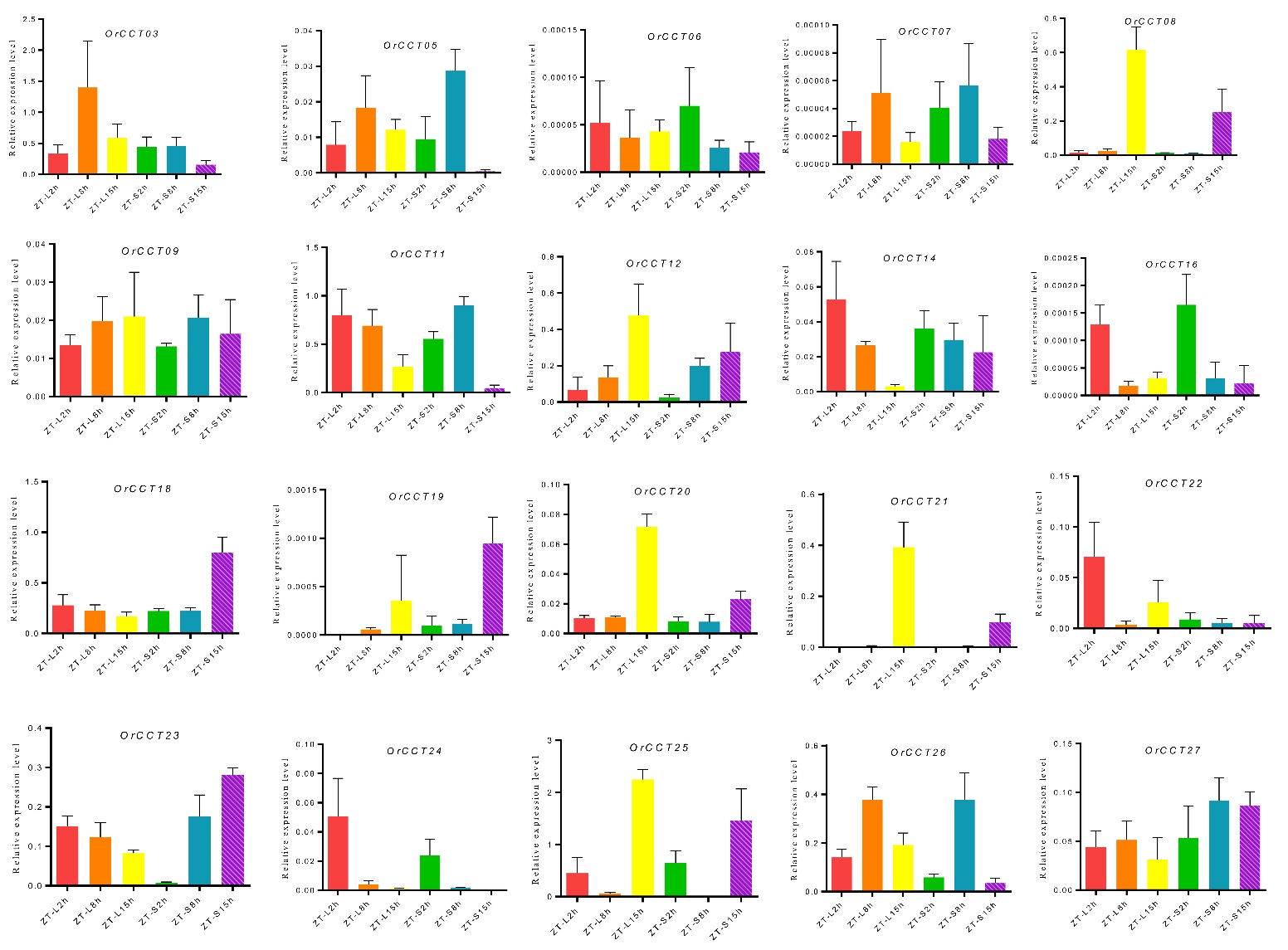


**Supplementary Figure 1.** The qRT-PCR validation of 20 *OrCCT* TFs with rhythmic expression patterns shown by RNA-seq analysis.

**Supplementary Table 1.** Primer pairs used in this study.

| **Gene name** | **Forward primer** | **Reverse primer** |
| --- | --- | --- |
| *Ubi*-RT | AACCAGCTGAGGCCCAAGA | ACGATTGATTTAACCAGTCCATGA |
| *Ehd1*_RT | GTTGCCAGTCATCTGCAGAA | TTCGGATGAGTAATCGTTCAG |
| *Hd3a*_RT | AGCCCAAGTGACCCTAACCT | GTTGTAGAGCTCGGCGAAGT |
| *RFT1*_RT | TGTCAGCTATGGTGCAAGGA | GAGCATCCGGGTCTACCAT |
| *OsGI*_RT | TGGAGAAAGGTTGTGGATGC | GATAGACGGCACTTCAGCAGAT |
| *OrCCT01*_RT | CAACAAGAAGATCACTTACGCG | GTCCTTGCGGTTGTAATTGTAG |
| *OrCCT02*_RT | GCATCCTCATCGTGTTCAGA | GCCGTTTCGTCGTTTATGTT |
| *OrCCT03*_RT | GCACCCGACTGTTCTCGAAG | GGCTCCTAACAAACCTTCCCT |
| *OrCCT04*_RT | TTAGTGCAGCGTCAACACCT | ACGATTCCGTTCTGAACATCA |
| *OrCCT05*_RT | CAAGGAGGTGAAGCTGGACT | AGGAGCTGTACGACGGTTTG |
| *OrCCT06*_RT | ATCCACTCGGCGAACCCGCT | CGCTTCTCCCTGTACCGCAT |
| *OrCCT07*_RT | CTGAAACAAGGCCGAGGGT | GCGGAGCAATGGACTGTTGA |
| *OrCCT08*_RT | CGACGCCATACAAGAACCCT | TCTGCCTTTTATGCCCAGCA |
| *OrCCT09*_RT | GCTCAACTACGAGGCGATCA | CCCGAGTAATGGTGGTCGTT |
| *OrCCT10*_RT | CATACGAACAGCGGACCGA | CTAGGCCGTCATCGCAGAAA |
| *OrCCT11*_RT | GAGACGATGCCACAGTTCCA | TGTGGGCTGTCAATCTCCAC |
| *OrCCT12*_RT | CCCAAGATTCAGAACTCGCC | ACCATTCTCATAACCAGGCGT |
| *OrCCT13*_RT | CCTCGTCGGAATCGGAGTTA | GTTCGTCCCTGCTTCACACT |
| *OrCCT14*_RT | ACGAGGGGTTCGACGATCT | GATGGAGGCGAAGTCCATGT |
| *OrCCT15*_RT | CACCATTTGGGCAGTTGCAG | TTTGATCCGTGGCTTGGTGA |
| *OrCCT16*_RT | ACTCCGACCCTTACCTCGAC | GCGTACCGGATGGTCTTCT |
| *OrCCT17*_RT | ATGGATACTGCGCCTACAGC | CACAGCTCGTAGTCCTCGTC |
| *OrCCT18*_RT | TCAGTTCTGGGTGCCTCAAC | TCAACCCAAACGCTGTCTCA |
| *OrCCT19*_RT | CCCTCAGCCTCAACTACGAG | TCTCCCTGTACCTCGACACC |
| *OrCCT20*_RT | TCAGCAACAGCATATCTTTCTCATCA | CTGGAATTTGGCATATCTATCACCG |
| *OrCCT21*_RT | CTCTGATGAGCAGCCCAAGC | CTGGACCTAGCTTGCCTCAC |
| *OrCCT22*_RT | GAAGCCTCAACCCTACATGC | TTCTTCCGCTTCTCCCTGTA |
| *OrCCT23*_RT | CCAACTCACCCTCTCGTTCC | GCAGATGACGATGCTCCAGA |
| *OrCCT24*_RT | GCTTGAACCCAAACACGG | GGGCCTCATCTCGGCATA |
| *OrCCT25*_RT | GAGAATCAGAGTTCGCCGGA | GCCAAAATCACCATGCCCAG |
| *OrCCT26*_RT | CCTATGGCAGCATGTGTGGA | TGAGCTCTGCGCTTGAGTG |
| *OrCCT27*_RT | CTGCTGCTGAAGCTCGACTA | TTGTACCGCCTCACCTTCTC |
| *OrCCT28*_RT | TCCAGAAGACCATCCGCTAC | GAAGGAGAGCCAGAACTTGG |
| *OrCCT29*_RT | CGATCAGAGGCCATGTACC | GCCTTTCACCCTCTTCCTC |
| *OrCCT30*_RT | GTGAGTATGGACATTACCGACT | GCTCTTGGATGACCTCAAGTA |
| *OrCCT31*_RT | CGGATTGACAAATGACGGGC | CATGGCGATCGTTTTGCTGT |
| *OrCCT32*_RT | TCAGTGGCAGTGTCTGTGAC | GGTGGCGTAAACCATCAGGA |
| *OrCCT33*_RT | AGAAGATCAAGGTGAGTACGCT | CTGGATGCCTCGCAGTAGTC |
| *OrCCT34*_RT | ATGTCCTTCTGTGGGAGCAC | CTCCGCTTCCTCTTCTCCTT |
| *OrCCT35*_RT | TCAAAGGGGACTGCAACAGG | TGGCCGTTGAGCTGATGATT |
| *OrCCT36*_RT | CCAACTGTGGTGAAAGCAGC | TCGATCTTCCTCGTGATGCG |
| *OrCCT37*_RT | GCTGCTGCAATCATCCCAAC | ACAGGTGGAGATTCATGCCC |
| *OrCCT38*_RT | CGCTAGAGTTGCCTCCATGT | CGCTTATAACTTGCGGTGGC |
| *OrCCT39*_RT | TGCAGATTTTACCGCTCCCT | TCATGATCCTGTGTTGCCCG |
| *OrCCT40*_RT | CACCATTCGAGCCGGTCTTC | GCGTAGGTGATCCTCTTGCC |
| *OrCCT41*_RT | GGTGCCGTCTTACCAGTCT | TTTCGCTTGCTCCGGTACTT |
| OX_*OrCCT24* | CATAAGCTTTATCCGTTCATGTCGATGGGA | CCGGTACCCTATCTGAACCATTGTCCAAGC |

**Supplementary Table 2.** Properties and locations of the CCT TFs in *O.rufipogon.*

| **Gene name** | ***Locus ID*** | **Protein (aa)** | **Chr.** | **Molecular Weight (D)** | **Theoretical Pi** | **Subfamily** | **Subcellular localization** |
| --- | --- | --- | --- | --- | --- | --- | --- |
| *OrCCT01* | RUF003834.t1 | 335 | 1 | 35780.84 | 7.12 | CMF | chloroplast thylakoid membrane |
| *OrCCT02* | RUF004918.t1 | 452 | 2 | 49108.72 | 5.08 | COL | extracellular space |
| *OrCCT03* | RUF005147.t2 | 483 | 2 | 51107.67 | 8.92 | CMF | chloroplast outer membrane |
| *OrCCT04* | RUF005151.t1 | 261 | 2 | 27395.82 | 8.35 | CMF | nucleus |
| *OrCCT05* | RUF005334.t1 | 345 | 2 | 35247.62 | 6.37 | COL | extracellular space |
| *OrCCT06* | RUF007335.t1 | 333 | 2 | 34949.93 | 5.11 | COL | nucleus |
| *OrCCT07* | RUF007394.t1 | 558 | 2 | 61721.08 | 5.71 | PRR | nucleus |
| *OrCCT08* | RUF008090.t1 | 408 | 2 | 44208.03 | 4.99 | COL | nucleus |
| *OrCCT09* | RUF008152.t1 | 453 | 2 | 49240.93 | 5.95 | COL | nucleus |
| *OrCCT10* | RUF009169.t1 | 412 | 3 | 45064.9 | 4.42 | CMF | nucleus |
| *OrCCT11* | RUF010172.t1 | 768 | 3 | 84107.03 | 6.03 | PRR | nucleus |
| *OrCCT12* | RUF010626.t1 | 405 | 3 | 43697.51 | 5.37 | COL | nucleus |
| *OrCCT13* | RUF011951.t1 | 1569 | 3 | 171328.16 | 4.97 | CMF | nucleus |
| *OrCCT14* | RUF012133.t1 | 381 | 3 | 41214.96 | 5.37 | COL | mitochondrion |
| *OrCCT15* | RUF012541.t1 | 1149 | 3 | 130495.42 | 7.1 | CMF | extracellular space |
| *OrCCT16* | RUF015130.t1 | 294 | 4 | 30449.58 | 4.97 | COL | nucleus |
| *OrCCT17* | RUF018658.t2 | 317 | 5 | 34210.35 | 5.43 | CMF | nucleus |
| *OrCCT18* | RUF019590.t1 | 309 | 5 | 34134.83 | 4.8 | CMF | nucleus |
| *OrCCT19* | RUF020670.t1 | 448 | 6 | 47997.5 | 5.08 | COL | chloroplast outer membrane |
| *OrCCT20* | RUF020733.t1 | 408 | 6 | 44360.88 | 4.09 | COL | nucleus |
| *OrCCT21* | RUF020864.t2 | 409 | 6 | 43935.87 | 5.08 | COL | nucleus |
| *OrCCT22* | RUF022167.t1 | 372 | 6 | 39204.13 | 6.16 | COL | nucleus |
| *OrCCT23* | RUF022479.t1 | 920 | 6 | 100978.19 | 5.44 | CMF | nucleus |
| *OrCCT24* | RUF023647.t1 | 258 | 7 | 27249.58 | 6.08 | CMF | nucleus |
| *OrCCT25* | RUF025415.t1 | 381 | 7 | 40357.86 | 5.04 | COL | nucleus |
| *OrCCT26* | RUF025635.t1 | 743 | 7 | 79900.06 | 8.73 | PRR | nucleus |
| *OrCCT27* | RUF025810.t1 | 305 | 8 | 31701.31 | 9.14 | CMF | chloroplast |
| *OrCCT28* | RUF026562.t1 | 301 | 8 | 29758.81 | 5.57 | COL | nucleus |
| *OrCCT29* | RUF028100.t1 | 493 | 8 | 52088.98 | 5.99 | COL | nucleus |
| *OrCCT30* | RUF028498.t2 | 336 | 9 | 36082.02 | 5.37 | COL | nucleus |
| *OrCCT31* | RUF029967.t2 | 472 | 9 | 50590.16 | 6.34 | COL | nucleus |
| *OrCCT32* | RUF030104.t1 | 624 | 9 | 69608.71 | 6.64 | PRR | nucleus |
| *OrCCT33* | RUF031915.t1 | 392 | 10 | 40384.29 | 4.73 | CMF | nucleus |
| *OrCCT34* | RUF032545.t1 | 298 | 10 | 30107.41 | 7.04 | CMF | nucleus |
| *OrCCT35* | RUF033028.t1 | 624 | 11 | 68476.25 | 8.43 | PRR | nucleus |
| *OrCCT36* | RUF034529.t1 | 1121 | 11 | 130415.08 | 8.87 | CMF | nucleus |
| *OrCCT37* | RUF035362.t1 | 224 | 12 | 24669.79 | 7.72 | CMF | nucleus |
| *OrCCT38* | RUF035363.t1 | 841 | 12 | 92021.56 | 5.59 | CMF | nucleus |
| *OrCCT39* | RUF035382.t1 | 784 | 12 | 85976.88 | 5.76 | CMF | nucleus |
| *OrCCT40* | RUF035384.t1 | 83 | 12 | 9689.86 | 11.44 | CMF | chloroplast |
| *OrCCT41* | RUF036195.t1 | 166 | 12 | 18084.75 | 5.27 | CMF | nucleus |

**Supplementary Table 3.** The sequence information for each motif of OrCCT TFs in *O. rufipogon.*

| **Motif^a^** | **Width** | **E value** | **Best possible match** |
| --- | --- | --- | --- |
| 1 | 46 | 7.5e-966 | [AG]EERE[AE][RA][LIV]M[RK][YF][RK]EKRKNR[NR]F[DE]KKIRY[AE][SCV]RKA[LY]A[DE][TS]RPR[VI][KR]G[RQ]F[AV][KR]R |
| 2 | 36 | 1.7E-268 | [PRA]AAV[YT]CRADAA[AR]LC[ALV]ACD[AR][DH][VI]HSAN[PA]L[AS]RRHER[VAL]P[LV] |
| 3 | 91 | 6.8E-155 | [KQH][AV][WI][DGNQT][VCIY][LM][EKNR][DEC][EKLMR][AQS][NFGY][AEHNS][IF]D[LI][VI]L[TA]EV[VDET][ML]P[GLRTV][LMSV][SK][GC][IF][EGKLS][LM]L[SK][RKTY]I[MATV][AENRS][HNS][DKN][EIA][CL][KR][NDH]IP[VI]IMMS[SN][NQR]D[SAE][MVI][GS][TMV]V[FLV][KR]C[LM][SLQR][KL]GA[VA][DE][FY]LVKP[ILV]R[KM]NEL[KRL]NLW[QT]HVWR[RK][CHQR][HALR][SM] |
| 4 | 39 | 1.6E-48 | C[HQT][HN]CG[IET][SN][AES][KAD][AMS]TP[MA]MR[RH][GA]P[DAN]G[PT][RK][TS][LF]CNACGLMWAN[KS][GV][TLM][LGM]R |
| 5 | 100 | 1.7E-40 | KCPHQSTGFPDYAKACTECDSGLTSCERVDR[AI][ST]QDHDILSGSSIYSKNKDLYSYSDISVFS[EV]THKDFAEPLELPPCGKDDEAPPDLAAQLHCNSCKDVMM |
| 6 | 100 | 2.9E-38 | KLLVEWTKGKNLIISSAAHTASEIRGPYDAINLSSYLLGLSTQRAKAALSVNCRSLISKALRKKHF[FY]KKTIRID[GR]LLPNKQLNSANFKLADWIGWDPMPH |
| 7 | 100 | 2.4E-36 | EHTPCGPETAL[IT]AFLYDKGSIDTTSKTDELAKQNSNSLEGDVAKIHEQLLNYSYASGEVEISLTRSEKRTKKLRSQHPIYVPFLGFLKSVSFKKKASKGW |
| 8 | 100 | 9.5E-35 | HHRRHPFLQYTRITLSLDSAAACASALAPSAARLLRTYDI[IV]AARPLTQAAFDHLCQATFDHLDIVSIDFSHKLPFRLKLPMLKLALQRGLHLEIAYSPLI |
| 9 | 90 | 5.5E-31 | [HQ]HSVATNLDNPGNNETVMAHDVQTAAVSSFDLKGIEKHVESLHDAMELDGTESSKMNLIADFTAPLSSDD[NS]LVCYAIPCSMELSDTSVVN |
| 10 | 92 | 8.5E-30 | CDRC[AV][GS]QPA[AM]VRCL[EV]EN[AT]SLCQNCDWNGH[GS]A[AG]SSAAGHKRQTINCYSGCPSS[AS]ELS[KR]IW[ST]F[SV][MS]DIP[NT]VA[AP]EPNCE[EQ]GI[NS]MMSI[NS]D[NS][DG]V[NS]N[HQ][CD] |

a: Numbers correspond to the motifs in Figure 1C

**Supplementary Table 4.** The information of orthologous CCT genes between *O. rufipogon* and *O. sativa.*

| **Locus_RUF** | **Gene name** | **Locus_SAT** | **Gene name** | **Homology**  **%** | **Annotation^a^** |
| --- | --- | --- | --- | --- | --- |
| RUF003834.t1 | *OrCCT01* | LOC_Os01g61900.1 | *OsCCT01* | 99.4 | regulation of flower development; ribonuclease P activity; RNA phosphodiester bond hydrolysis, endonucleolytic |
| RUF004918.t1 | *OrCCT02* | LOC_Os02g01990.1 | *OsCCT02* | 99.07 | nucleus location |
| RUF005147.t2 | *OrCCT03* | LOC_Os02g05470.1 | *OsCCT03* | 99.79 | regulation of transcription, DNA-templated; glycosyltransferase activity |
| RUF005151.t1 | *OrCCT04* | LOC_Os02g05510.1 | *OsCCT04* | 99.62 | sequence-specific DNA binding; regulation of transcription, DNA-templated; zinc ion binding; DNA-binding transcription factor activity |
| RUF005334.t1 | *OrCCT05* | LOC_Os02g08150.1 | *OsCCT05* | 99.14 | zinc ion binding |
| RUF007335.t1 | *OrCCT06* | LOC_Os02g39710.1 | *OsCCT06* | 99.4 | zinc ion binding |
| RUF007394.t1 | *OrCCT07* | LOC_Os02g40510.1 | *OsCCT07* | 100 | rhythmic process; protein binding; phosphorelay signal transduction system; motor activity; ATP binding |
| RUF008090.t1 | *OrCCT08* | LOC_Os02g49230.2 | *OsCCT08* | 99.26 | zinc ion binding |
| RUF008152.t1 | *OrCCT09* | LOC_Os02g49880.1 | *OsCCT09* | 100 | zinc ion binding; regulation of transcription, DNA-templated; DNA-binding transcription factor activity; DNA binding |
| RUF009169.t1 | *OrCCT10* | LOC_Os03g04620.1 | *OsCCT10* | 99.76 | regulation of flower development; ribonuclease P activity; RNA phosphodiester bond hydrolysis, endonucleolytic |
| RUF010172.t1 | *OrCCT11* | LOC_Os03g17570.3 | *OsCCT11* | 99.87 | rhythmic process; kinase activity; phosphorelay signal transduction system; phosphorylation |
| RUF010626.t1 | *OrCCT12* | LOC_Os03g22770.1 | *OsCCT12* | 99.26 | zinc ion binding |
| RUF011951.t1 | *OrCCT13* | LOC_Os03g47970.1 | *OsCCT13* | 98.77 | uncharacterized protein |
| RUF012133.t1 | *OrCCT14* | LOC_Os03g50310.1 | *OsCCT14* | 100 | zinc ion binding; regulation of transcription, DNA-templated |
| RUF012541.t1 | *OrCCT15* | LOC_Os03g52450.1 | *OsCCT15* | 99.75 | zinc ion binding; regulation of transcription, DNA-templated |
| RUF015130.t1 | *OrCCT16* | LOC_Os04g42020.1 | *OsCCT16* | 88.02 | zinc ion binding |
| RUF018658.t2 | *OrCCT17* | LOC_Os05g38990.1 | *OsCCT17* | 96.49 | regulation of flower development; ribonuclease P activity; RNA phosphodiester bond hydrolysis, endonucleolytic; |
| RUF019590.t1 | *OrCCT18* | LOC_Os05g51690.1 | *OsCCT18* | 99.68 | regulation of flower development |
| RUF020670.t1 | *OrCCT19* | LOC_Os06g15330.1 | *OsCCT20* | 98.89 | zinc ion binding; regulation of transcription, DNA-templated; DNA-binding transcription factor activity; DNA binding |
| RUF020733.t1 | *OrCCT20* | LOC_Os06g16370.1 | *OsCCT21* | 95.83 | zinc ion binding; positive regulation of short-day photoperiodism, flowering; negative regulation of long-day photoperiodism, flowering; negative regulation of transcription, DNA-templated |
| RUF020864.t2 | *OrCCT21* | LOC_Os06g19444.1 | *OsCCT22* | 100 | zinc ion binding |

Continued

| **Locus_RUF** | **Gene name** | **Locus_SAT** | **Gene name** | **Homology**  **%** | **Annotation^a^** |
| --- | --- | --- | --- | --- | --- |
| RUF022167.t1 | *OrCCT22* | LOC_Os06g44450.1 | *OsCCT23* | 100 | zinc ion binding |
| RUF022479.t1 | *OrCCT23* | LOC_Os06g48534.1 | *OsCCT24* | 99.66 | ATP binding; regulation of meristem development; regulation of alternative mRNA splicing, via spliceosome; defense response |
| RUF023647.t1 | *OrCCT24* | LOC_Os07g15770.1 | *OsCCT26* | 98.84 | negative regulation of long-day photoperiodism, flowering; protein binding; DNA binding; zinc ion binding; regulation of flower development; flower development |
| RUF025415.t1 | *OrCCT25* | LOC_Os07g47140.1 | *OsCCT27* | 100 | zinc ion binding |
| RUF025635.t1 | *OrCCT26* | LOC_Os07g49460.1 | *OsCCT28* | 98.79 | rhythmic process; phosphorelay signal transduction system |
| RUF026562.t1 | *OrCCT28* | LOC_Os08g15050.1 | *OsCCT29* | 98.68 | zinc ion binding |
| RUF028100.t1 | *OrCCT29* | LOC_Os08g42440.1 | *OsCCT30* | 98.78 | zinc ion binding |
| RUF028498.t2 | *OrCCT30* | LOC_Os09g06464.1 | *OsCCT31* | 100 | negative regulation of short-day photoperiodism, flowering; zinc ion binding; DNA binding |
| RUF029967.t2 | *OrCCT31* | LOC_Os09g33550.1 | *OsCCT32* | 100 | zinc ion binding; protein ubiquitination; DNA integration; nucleic acid binding |
| RUF030104.t1 | *OrCCT32* | LOC_Os09g36220.1 | *OsCCT33* | 99.68 | rhythmic process; kinase activity; phosphorelay signal transduction system; phosphorylation |
| RUF031915.t1 | *OrCCT33* | LOC_Os10g32900.1 | *OsCCT34* | 93.64 | regulation of flower development |
| RUF032545.t1 | *OrCCT34* | LOC_Os10g41100.1 | *OsCCT35* | 99.66 | negative regulation of long-day photoperiodism, flowering; protein binding; DNA binding; zinc ion binding; regulation of flower development; flower development |
| RUF033028.t1 | *OrCCT35* | LOC_Os11g05930.1 | *OsCCT38* | 99.36 | rhythmic process; kinase activity; phosphorelay signal transduction system; phosphorylation |
| RUF035362.t1 | *OrCCT37* | LOC_Os12g01100.1 | *OsCCT40* | 100 | regulation of flower development; ribonuclease P activity; RNA phosphodiester bond hydrolysis, endonucleolytic; |
| RUF035363.t1 | *OrCCT38* | LOC_Os11g01074.4 | *OsCCT36* | 99.59 | ribonuclease P activity; RNA phosphodiester bond hydrolysis, endonucleolytic; |
| RUF035382.t1 | *OrCCT39* | LOC_Os11g01074.4 | *OsCCT36* | 97.19 | regulation of flower development; ribonuclease P activity; RNA phosphodiester bond hydrolysis, endonucleolytic; RNA binding |
| RUF036195.t1 | *OrCCT41* | LOC_Os12g16160.1 | *OsCCT41* | 99.4 | regulation of flower development; ribonuclease P activity; RNA phosphodiester bond hydrolysis, endonucleolytic |
| RUF025810.t1 | *OrCCT27* | absent in Nipponbare | — |  | regulation of transcription, DNA-templated |
| RUF034529.t1 | *OrCCT36* | absent in Nipponbare | — |  | uncharacterized protein |
| RUF035384.t1 | *OrCCT40* | absent in Nipponbare | — |  | regulation of flower development; ribonuclease P activity; RNA phosphodiester bond hydrolysis, endonucleolytic; |

a: Annotated by PANNZER (http://ekhidna2.biocenter.helsinki.fi/sanspanz/)

**Supplementary Table 5.** The estimated ages of the duplicated CCT gene pairs in *O. rufipogon* and *O. sativa.*

| *O. rufipogon* | | | | | *O. sativa* | | | | | Time (MYA) |
| --- | --- | --- | --- | --- | --- | --- | --- | --- | --- | --- |
| Duplicated gene pairs | | Ka | Ks | ω | Duplicated gene pairs | | Ka | Ks | ω |  |
| *OrCCT01* | *OrCCT17* | 0.31 | 1.02 | 0.3 | *OsCCT01* | *OsCCT17* | 0.34 | 1.03 | 0.33 | 78.36 ~ 79.51 |
| *OrCCT04* | *OrCCT23* | 0.21 | 0.85 | 0.24 | *OsCCT04* | *OsCCT24* | 0.22 | 0.8 | 0.27 | 61.81 ~ 65.48 |
| *OrCCT05* | *OrCCT22* | 0.24 | 0.41 | 0.58 | *OsCCT05* | *OsCCT23* | 0.22 | 0.44 | 0.5 | 31.18 ~ 33.51 |
| *OrCCT06* | *OrCCT16* | 0.16 | 0.3 | 0.55 | *OsCCT06* | *OsCCT16* | 0.17 | 0.32 | 0.54 | 23.05 ~ 24.42 |
| *OrCCT08* | *OrCCT21* | 0.13 | 0.93 | 0.14 | *OsCCT08* | *OsCCT22* | 0.13 | 0.93 | 0.14 | 71.75 ~ 71.84 |
| *OrCCT09* | *OrCCT19* | 0.26 | 0.47 | 0.55 | *OsCCT09* | *OsCCT20* | 0.26 | 0.46 | 0.57 | 35.41 ~ 36.25 |
| *OrCCT10* | *OrCCT33* | 0.49 | 1.16 | 0.42 | *OsCCT10* | *OsCCT34* | 0.4 | 1.14 | 0.35 | 87.69 ~ 89.31 |
| *OrCCT11* | *OrCCT26* | 0.22 | 1.02 | 0.22 | *OsCCT11* | *OsCCT28* | 0.24 | 1.05 | 0.22 | 78.49 ~ 81.12 |
| *OrCCT12* | *OrCCT25* | 0.33 | 0.75 | 0.43 | *OsCCT12* | *OsCCT27* | 0.33 | 0.76 | 0.44 | 57.84 ~ 58.64 |
| *OrCCT29* | *OrCCT31* | 0.28 | 0.75 | 0.37 | *OsCCT30* | *OsCCT32* | 0.29 | 0.77 | 0.38 | 57.65 ~ 59.31 |
| *OrCCT38* | *OrCCT39* | 0.46 | 0.73 | 0.63 | *OsCCT36* | Absent | — | — | — | ~ 56.06 |
| Absent^a^ | *OrCCT37* | — | — | — | *OsCCT37* | *OsCCT40* | 0 | 0.01 | 0.32 | ~ 0.70 |
| *OrCCT03* | Absent | — | — | — | *OsCCT03* | *OsCCT25* | 0.28 | 0.61 | 0.46 | ~ 46.88 |

**Supplementary Table 6.** The loci of CCT TFs identified in this study.

| **Locus ID** | **Gene name** | **Size (aa)** | **Subfamily** | **Locus ID** | **Gene name** | **Size (aa)** | **Subfamily** |
| --- | --- | --- | --- | --- | --- | --- | --- |
| ***O. rufipogon* acc. R1** | | | | | | | |
| RUF003834.t1 | *OrCCT01* | 334 | CMF | RUF022167.t1 | *OrCCT22* | 215 | COL |
| RUF004918.t1 | *OrCCT02* | 451 | COL | RUF022479.t1 | *OrCCT23* | 919 | CMF |
| RUF005147.t2 | *OrCCT03* | 482 | CMF | RUF023647.t1 | *OrCCT24* | 257 | CMF |
| RUF005151.t1 | *OrCCT04* | 260 | CMF | RUF025415.t1 | *OrCCT25* | 380 | COL |
| RUF005334.t1 | *OrCCT05* | 349 | COL | RUF025635.t1 | *OrCCT26* | 742 | PRR |
| RUF007335.t1 | *OrCCT06* | 332 | COL | RUF025810.t1 | *OrCCT27* | 304 | CMF |
| RUF007394.t1 | *OrCCT07* | 557 | PRR | RUF026562.t1 | *OrCCT28* | 300 | COL |
| RUF008090.t1 | *OrCCT08* | 407 | COL | RUF028100.t1 | *OrCCT29* | 492 | COL |
| RUF008152.t1 | *OrCCT09* | 452 | COL | RUF028498.t2 | *OrCCT30* | 335 | COL |
| RUF009169.t1 | *OrCCT10* | 411 | CMF | RUF029967.t2 | *OrCCT31* | 471 | COL |
| RUF010172.t1 | *OrCCT11* | 767 | PRR | RUF030104.t1 | *OrCCT32* | 623 | PRR |
| RUF010626.t1 | *OrCCT12* | 404 | COL | RUF031915.t1 | *OrCCT33* | 391 | CMF |
| RUF011951.t1 | *OrCCT13* | 1568 | CMF | RUF032545.t1 | *OrCCT34* | 297 | CMF |
| RUF012133.t1 | *OrCCT14* | 380 | COL | RUF033028.t1 | *OrCCT35* | 623 | PRR |
| RUF012541.t1 | *OrCCT15* | 1148 | CMF | RUF034529.t1 | *OrCCT36* | 1120 | CMF |
| RUF015130.t1 | *OrCCT16* | 293 | COL | RUF035362.t1 | *OrCCT37* | 223 | CMF |
| RUF018658.t2 | *OrCCT17* | 316 | CMF | RUF035363.t1 | *OrCCT38* | 840 | CMF |
| RUF019590.t1 | *OrCCT18* | 308 | CMF | RUF035382.t1 | *OrCCT39* | 783 | CMF |
| RUF020670.t1 | *OrCCT19* | 447 | COL | RUF035384.t1 | *OrCCT40* | 82 | CMF |
| RUF020733.t1 | *OrCCT20* | 407 | COL | RUF036195.t1 | *OrCCT41* | 165 | CMF |
| RUF020864.t2 | *OrCCT21* | 408 | COL |  |  |  |  |
| ***O. nivara*** | | | | | | | |
| Oniva01g32960.1 | *OnCCT01* | 287 | CMF | Oniva06g13380.1 | *OnCCT22* | 408 | COL |
| Oniva01g41360.1 | *OnCCT02* | 334 | CMF | Oniva06g27450.1 | *OnCCT23* | 370 | COL |
| Oniva02g00710.1 | *OnCCT03* | 323 | CMF | Oniva06g28780.1 | *OnCCT24* | 1812 | CMF |
| Oniva02g03780.1 | *OnCCT04* | 473 | CMF | Oniva07g01960.1 | *OnCCT25* | 642 | CMF |
| Oniva02g03810.1 | *OnCCT05* | 328 | CMF | Oniva07g25200.1 | *OnCCT26* | 380 | COL |
| Oniva02g06890.1 | *OnCCT06* | 349 | COL | Oniva07g27300.3 | *OnCCT27* | 755 | PRR |
| Oniva02g26540.1 | *OnCCT07* | 332 | COL | Oniva08g01360.1 | *OnCCT28* | 351 | CMF |
| Oniva02g33840.1 | *OnCCT08* | 474 | COL | Oniva08g08540.1 | *OnCCT29* | 302 | COL |
| Oniva02g34840.1 | *OnCCT09* | 452 | COL | Oniva08g24870.1 | *OnCCT30* | 483 | COL |
| Oniva03g02870.1 | *OnCCT10* | 411 | CMF | Oniva09g01720.1 | *OnCCT31* | 335 | COL |
| Oniva03g14290.2 | *OnCCT11* | 892 | PRR | Oniva09g12440.2 | *OnCCT32* | 585 | PRR |
| Oniva03g18400.1 | *OnCCT12* | 403 | COL | Oniva09g17070.1 | *OnCCT33* | 462 | COL |
| Oniva03g31130.1 | *OnCCT13* | 319 | CMF | Oniva09g18470.1 | *OnCCT34* | 623 | PRR |
| Oniva03g32910.1 | *OnCCT14* | 421 | COL | Oniva10g12430.1 | *OnCCT35* | 450 | CMF |
| Oniva03g34920.1 | *OnCCT15* | 271 | CMF | Oniva10g12560.1 | *OnCCT36* | 528 | CMF |
| Oniva04g15770.1 | *OnCCT16* | 331 | COL | Oniva10g20970.1 | *OnCCT37* | 297 | CMF |
| Oniva04g24360.1 | *OnCCT17* | 314 | CMF | Oniva11g03700.2 | *OnCCT38* | 742 | PRR |
| Oniva05g30050.1 | *OnCCT18* | 308 | CMF | Oniva12g00060.1 | *OnCCT39* | 147 | CMF |
| Oniva06g00130.1 | *OnCCT19* | 229 | COL | Oniva12g06600.1 | *OnCCT40* | 349 | CMF |
| Oniva06g11650.1 | *OnCCT20* | 453 | COL | Oniva12g10390.1 | *OnCCT41* | 223 | CMF |
| Oniva06g12400.1 | *OnCCT21* | 408 | COL |  |  |  |  |
| ***O. sativa* spp. *japonica acc. Nipponbare*** | | | | | | | |
| LOC_Os01g61900.1 | *OsCCT01* | 335 | CMF | LOC_Os02g49230.2 | *OsCCT08* | 480 | COL |
| Continued |  |  |  |  |  |  |  |
| **Locus ID** | **Gene name** | **Size (aa)** | **Subfamily** | **Locus ID** | **Gene name** | **Size (aa)** | **Subfamily** |
| LOC_Os02g01990.1 | *OsCCT02* | 323 | CMF | LOC_Os02g49880.1 | *OsCCT09* | 452 | COL |
| LOC_Os02g05470.1 | *OsCCT03* | 482 | CMF | LOC_Os03g22770.1 | *OsCCT12* | 403 | COL |
| LOC_Os02g05510.1 | *OsCCT04* | 328 | CMF | LOC_Os03g50310.1 | *OsCCT14* | 421 | COL |
| LOC_Os03g04620.1 | *OsCCT10* | 411 | CMF | LOC_Os04g42020.1 | *OsCCT16* | 333 | COL |
| LOC_Os03g47970.1 | *OsCCT13* | 319 | CMF | LOC_Os06g01340.1 | *OsCCT19* | 223 | COL |
| LOC_Os03g52450.1 | *OsCCT15* | 271 | CMF | LOC_Os06g15330.1 | *OsCCT20* | 448 | COL |
| LOC_Os05g38990.1 | *OsCCT17* | 324 | CMF | LOC_Os06g16370.1 | *OsCCT21* | 395 | COL |
| LOC_Os05g51690.1 | *OsCCT18* | 308 | CMF | LOC_Os06g19444.1 | *OsCCT22* | 408 | COL |
| LOC_Os06g48534.1 | *OsCCT24* | 292 | CMF | LOC_Os06g44450.1 | *OsCCT23* | 371 | COL |
| LOC_Os06g48610.1 | *OsCCT25* | 466 | CMF | LOC_Os07g47140.1 | *OsCCT27* | 380 | COL |
| LOC_Os07g15770.1 | *OsCCT26* | 287 | CMF | LOC_Os08g15050.1 | *OsCCT29* | 303 | COL |
| LOC_Os10g32900.1 | *OsCCT34* | 449 | CMF | LOC_Os08g42440.1 | *OsCCT30* | 488 | COL |
| LOC_Os10g41100.1 | *OsCCT35* | 297 | CMF | LOC_Os09g06464.1 | *OsCCT31* | 335 | COL |
| LOC_Os11g01074.4 | *OsCCT36* | 564 | CMF | LOC_Os09g33550.1 | *OsCCT32* | 471 | COL |
| LOC_Os11g01100.1 | *OsCCT37* | 153 | CMF | LOC_Os02g40510.1 | *OsCCT07* | 518 | PRR |
| LOC_Os12g01080.1 | *OsCCT39* | 146 | CMF | LOC_Os03g17570.3 | *OsCCT11* | 767 | PRR |
| LOC_Os12g01100.1 | *OsCCT40* | 153 | CMF | LOC_Os07g49460.1 | *OsCCT28* | 742 | PRR |
| LOC_Os12g16160.1 | *OsCCT41* | 220 | CMF | LOC_Os09g36220.1 | *OsCCT33* | 623 | PRR |
| LOC_Os02g08150.1 | *OsCCT05* | 349 | COL | LOC_Os11g05930.1 | *OsCCT38* | 699 | PRR |
| LOC_Os02g39710.1 | *OsCCT06* | 332 | COL |  |  |  |  |
| ***O. sativa spp. indica acc. R498*** | | | | | | | |
| OsR498G0102285600.01.P01 | *IndCCT01* | 334 | CMF | OsR498G0612055300.01.P01 | *IndCCT21* | 447 | COL |
| OsR498G0202832000.01.P01 | *IndCCT02* | 225 | CMF | OsR498G0612090700.01.P01 | *IndCCT22* | 441 | COL |
| OsR498G0202971900.01.P01 | *IndCCT03* | 484 | CMF | OsR498G0612192000.01.P01 | *IndCCT23* | 408 | COL |
| OsR498G0202973700.01.P01 | *IndCCT04* | 328 | CMF | OsR498G0613085400.01.P02 | *IndCCT24* | 373 | COL |
| OsR498G0203084300.01.P02 | *IndCCT05* | 349 | COL | OsR498G0613248200.01.P01 | *IndCCT25* | 340 | CMF |
| OsR498G0204282700.01.P01 | *IndCCT06* | 332 | COL | OsR498G0613250500.01.P01 | *IndCCT26* | 466 | CMF |
| OsR498G0204327700.01.P01 | *IndCCT07* | 518 | PRR | OsR498G0713935400.01.P01 | *IndCCT27* | 257 | CMF |
| OsR498G0204681300.01.P06 | *IndCCT08* | 474 | COL | OsR498G0714919400.01.P01 | *IndCCT28* | 380 | COL |
| OsR498G0204708800.01.P01 | *IndCCT09* | 452 | COL | OsR498G0715020100.01.P01 | *IndCCT29* | 742 | PRR |
| OsR498G0305202800.01.P01 | *IndCCT10* | 411 | CMF | OsR498G0815087000.01.P01 | *IndCCT30* | 228 | CMF |
| OsR498G0305710000.01.P02 | *IndCCT11* | 767 | PRR | OsR498G0815539300.01.P01 | *IndCCT31* | 275 | COL |
| OsR498G0305948100.01.P04 | *IndCCT12* | 403 | COL | OsR498G0816533200.01.P01 | *IndCCT32* | 492 | COL |
| OsR498G0306812800.01.P01 | *IndCCT13* | 319 | CMF | OsR498G0916781000.01.P03 | *IndCCT33* | 144 | CMF |
| OsR498G0306913700.01.P01 | *IndCCT14* | 421 | COL | OsR498G0917780800.01.P01 | *IndCCT34* | 471 | COL |
| OsR498G0307002400.01.P02 | *IndCCT15* | 271 | CMF | OsR498G0917850800.01.P01 | *IndCCT35* | 623 | PRR |
| OsR498G0307144200.01.P01 | *IndCCT16* | 1148 | CMF | OsR498G1018867100.01.P01 | *IndCCT36* | 424 | CMF |
| OsR498G0408832700.01.P01 | *IndCCT17* | 331 | COL | OsR498G1019192800.01.P01 | *IndCCT37* | 297 | CMF |
| OsR498G0511005600.01.P01 | *IndCCT18* | 313 | CMF | OsR498G1119451500.01.P01 | *IndCCT38* | 699 | PRR |
| OsR498G0511456100.01.P01 | *IndCCT19* | 308 | CMF | OsR498G1120395000.01.P01 | *IndCCT39* | 164 | CMF |
| OsR498G0611471700.01.P01 | *IndCCT20* | 129 | CMF | OsR498G1221237400.01.P01 | *IndCCT40* | 166 | CMF |
| ***Brachypodium distachyon*** | | | | | | | |
| Bradi1g06907.2.p | *BdCCT01* | 1193 | CMF | Bradi3g03800.1.p | *BdCCT21* | 359 | CMF |
| Bradi1g09550.1.p | *BdCCT02* | 285 | CMF | Bradi3g03810.2.p | *BdCCT22* | 219 | CMF |
| Bradi1g11310.2.p | *BdCCT03* | 424 | COL | Bradi3g05800.1.p | *BdCCT23* | 380 | COL |
| Bradi1g12330.5.p | *BdCCT04* | 297 | CMF | Bradi3g10010.2.p | *BdCCT24* | 220 | CMF |
| Continued |  |  |  |  |  |  |  |
| **Locus ID** | **Gene name** | **Size (aa)** | **Subfamily** | **Locus ID** | **Gene name** | **Size (aa)** | **Subfamily** |
| Bradi1g16490.5.p | *BdCCT05* | 661 | PRR | Bradi3g13960.2.p | *BdCCT25* | 310 | CMF |
| Bradi1g18407.4.p | *BdCCT06* | 405 | CMF | Bradi3g19010.1.p | *BdCCT26* | 266 | COL |
| Bradi1g31280.1.p | *BdCCT07* | 367 | COL | Bradi3g28290.1.p | *BdCCT27* | 410 | CMF |
| Bradi1g33980.2.p | *BdCCT08* | 360 | CMF | Bradi3g41500.1.p | *BdCCT28* | 545 | COL |
| Bradi1g34060.1.p | *BdCCT09* | 449 | CMF | Bradi3g48447.1.p | *BdCCT29* | 326 | COL |
| Bradi1g43220.1.p | *BdCCT10* | 402 | COL | Bradi3g48880.1.p | *BdCCT30* | 522 | PRR |
| Bradi1g43670.1.p | *BdCCT11* | 379 | CMF | Bradi3g56260.2.p | *BdCCT31* | 369 | COL |
| Bradi1g43990.1.p | *BdCCT12* | 437 | COL | Bradi3g56490.1.p | *BdCCT32* | 394 | COL |
| Bradi1g52360.1.p | *BdCCT13* | 394 | COL | Bradi3g57000.2.p | *BdCCT33* | 449 | COL |
| Bradi1g62420.1.p | *BdCCT14* | 395 | CMF | Bradi4g15720.1.p | *BdCCT34* | 258 | CMF |
| Bradi1g65910.2.p | *BdCCT15* | 767 | PRR | Bradi4g24967.1.p | *BdCCT35* | 721 | PRR |
| Bradi1g75760.2.p | *BdCCT16* | 406 | CMF | Bradi4g36077.1.p | *BdCCT36* | 627 | PRR |
| Bradi2g14220.1.p | *BdCCT17* | 328 | CMF | Bradi4g45327.1.p | *BdCCT37* | 158 | CMF |
| Bradi2g22800.2.p | *BdCCT18* | 315 | CMF | Bradi4g45330.1.p | *BdCCT38* | 195 | CMF |
| Bradi2g54260.2.p | *BdCCT19* | 325 | CMF | Bradi5g14600.1.p | *BdCCT39* | 342 | COL |
| Bradi3g03770.1.p | *BdCCT20* | 471 | CMF |  |  |  |  |
